# RelBE-mediated stress responses reshape cellular morphology of *Weissella cibaria* under chloramphenicol exposure

**DOI:** 10.1128/spectrum.02518-25

**Published:** 2026-05-18

**Authors:** Han-Yang Wang, Wen-Liang Xiang, Ting Cai, Hao-Yu Zhu, Pei Shi, Qiao-Ni Xiong

**Affiliations:** 1 School of Food and Bioengineering, Xihua University 12598, Chengdu, China; 2 Food Microbiology Key Laboratory of Sichuan Province, Xihua University 12598, Chengdu, Sichuan, China; 3 United Graduate School of Agricultural Sciences, Kagoshima University 723141 , Kagoshima, Japan; University of Mississippi, University, Mississippi, USA

**Keywords:** *Weissella cibaria*, toxin-antitoxin system, RelBE, morphological regulation, persister cell, chloramphenicol

## Abstract

Morphological remodeling accompanies the emergence of persister states in some bacteria, yet the regulatory mechanisms underlying these changes remain poorly defined. *Weissella cibaria* is an emerging probiotic bacterium that exhibits pronounced phenotypic heterogeneity under antibiotic stress. Here, we show that exposure to chloramphenicol (CAP) activates the type II toxin-antitoxin module RelBE in *W. cibaria* and is associated with growth arrest and marked cellular elongation in a subpopulation of cells. RelBE consists of the mRNA endonuclease RelE and its cognate antitoxin RelB. Under non-stress conditions, RelBE is maintained at low levels as a non-toxic complex. CAP treatment induces the ClpXP protease, promoting RelB degradation, and transiently increasing free RelE. RelE activity coincides with coordinated transcriptional remodeling of pathways governing cell wall and membrane biogenesis, suppression of core cell division genes, and induction of biofilm-associated programs. These changes are accompanied by compromised cell wall integrity, membrane depolarization, enhanced aggregation, and inhibition of septation, collectively providing a mechanistic basis for the observed elongation phenotype. Rather than establishing a universal role for RelBE in persister formation, our results define how RelE activity reshapes cellular morphological architecture under antibiotic stress. This work links toxin-mediated mRNA cleavage to envelope remodeling, division blockade, and surface-associated adaptations, offering mechanistic insight into stress-induced morphological plasticity in a probiotic bacterium.

**IMPORTANCE:**

Probiotic bacteria frequently encounter antibiotic and industrial stresses that challenge their viability and functional stability. Understanding how these organisms adapt at the cellular level is therefore critical for both microbiology and applied biotechnology. This study reveals how the RelBE toxin-antitoxin (TA) module reshapes cellular architecture in the probiotic *Weissella cibaria* under chloramphenicol stress. We show that controlled activation of the RelE toxin coincided with coordinated remodeling of envelope biogenesis, repression of cell division programs, and enhancement of surface-associated behaviors, culminating in pronounced cell elongation. Rather than acting through a single pathway, RelBE engaged multiple cellular systems to generate stress-adaptive morphology. These findings provide a mechanistic insight into stress-induced phenotypic plasticity in beneficial bacteria and suggest that rational modulation of TA systems could be exploited to improve probiotic robustness and performance under adverse industrial and therapeutic conditions.

## INTRODUCTION

The discovery of antibiotics marked a significant milestone in the history of combating bacterial infections. However, complete eradication of bacterial populations is often difficult to achieve, in part because a small subpopulation of cells can transiently survive otherwise lethal treatments. These cells, commonly referred to as persistence, enter a reversible state of growth arrest and can repopulate once antibiotic pressure is relieved (1, 2). Importantly, persistence is a phenotypic state rather than a genetically fixed trait and arises stochastically within isogenic populations. Although persister bacteria are frequently associated with treatment failure and recurrent infections, their formation is now understood to result from complex, context-dependent physiological processes rather than from a single dedicated pathway.

In some bacterial species and under specific conditions, entry into persistence has been accompanied by pronounced morphological changes (3). These changes, when present, may include abnormal elongation, filamentation, or even transitions from spiral to coccoid forms (4). However, such alterations are neither universal nor obligatory features of persistence. Nevertheless, morphological remodeling provides a visually accessible window into the physiological reprogramming that accompanies dormancy and stress adaptation, and it remains poorly understood at the mechanistic level.

Toxin-antitoxin (TA) systems have long been implicated in stress responses and growth modulation, and early studies proposed a direct causal link between TA activation and persister formation. More recent work, however, has revealed substantial experimental pitfalls in this field and has emphasized that TA modules should not be viewed as the universal persister generators. Instead, TA systems are now regarded as components of broader regulatory networks whose biological impact depends on regulatory context, activation dynamics, and cellular state (5–7). Notwithstanding these revisions, controlled activation of certain toxins has been shown to recapitulate persister physiological and morphological phenotypes in defined experimental settings. For example, AapA1 induced a spiral-to-coccoid transition in *Helicobacter pylori* (8), MazF inhibited division and caused elongation in *Escherichia coli* (3), and HigB or PemK overexpression could induce marked cell elongation in *Weissella cibaria* or heterologous host *Escherichia coli* BL21 (4, 9). These studies highlight a reproducible association between toxin activity and morphological remodeling, while leaving the underlying regulatory mechanisms largely unresolved.

Chloramphenicol (CAP) is a broad-spectrum antibiotic widely used in aquaculture to control bacterial infections caused by pathogens such as *Enterobacter* and *Vibrio* spp. However, CAP treatment also perturbs beneficial members of the intestinal microbiota, necessitating the use of probiotics as supportive interventions. A critical but poorly understood issue is whether candidate probiotics can maintain viability and function under residual CAP pressure in the host intestine. *W. cibaria* is an emerging probiotic bacterium with documented antioxidant, immunomodulatory, and exopolysaccharide-producing capacities (10, 11), and has been shown to suppress *Edwardsiella ictaluri* in aquaculture settings (12). In our preliminary observations, *W. cibaria* exhibited a notable capacity to survive CAP exposure, accompanied by the emergence of persister cells, suggesting its potential suitability as a dietary probiotic during and after antibiotic treatment. When *W. cibaria* was exposed to CAP, the type II TA module RelBE became activated and was associated with pronounced phenotypic heterogeneity within isogenic populations. Among the most conspicuous features of this heterogeneity was a striking elongation of a subpopulation of cells. While RelBE has been implicated in metabolic reprogramming during stress adaptation, its contribution to morphological remodeling remains unknown.

Rather than attempting to establish a universal role for RelBE in persister formation, the present study addressed a more focused mechanistic question: how does RelE activity impinge on cellular systems governing morphology? Specifically, we systematically examined the effects of RelBE on cell wall biosynthesis, membrane integrity, cell division, and biofilm formation. By linking RelE-mediated mRNA cleavage to defined morphological and physiological outputs, this work clarified how toxin activity reshaped cellular architecture under antibiotic stress. These findings provided a mechanistic insight into stress-induced morphological plasticity and offer a framework for evaluating probiotic performance in antibiotic-impacted intestinal environments.

## MATERIALS AND METHODS

### Bacterial strains and culture conditions

The *W. cibaria* CGMCC 1.19376 (Xhu 414) was deposited in the Key Laboratory of Food Microbiology of Sichuan, Xihua University (Chengdu, China), and it was cultivated in De Man, Rogosa, and Sharpe (MRS) broth medium at 30°C before experiments unless otherwise stated. The *E. coli* BL21 (DE3) was cultivated in Luria-Bertani (LB) liquid medium at 37°C. For experiments involving toxin induction, cultivation temperatures were reduced to 25°C for *W. cibaria* and 30°C for recombinant *E. coli* to minimize non-physiological effects associated with excessive toxin accumulation.

### Determination of MIC and MBC of CAP

The minimum inhibitory concentration (MIC) of CAP against *W. cibaria* CGMCC 1.19376 was determined using a standard microbroth dilution assay in MRS medium (13). Briefly, 198 µL of MRS broth containing serial twofold dilutions of CAP (0–1,024 µg/mL) was dispensed into 96-well plates and inoculated with 2 µL of bacterial suspension (1 × 10⁶ CFU/mL). Plates were incubated at 30°C for 24 h, and OD₆₀₀ was measured. MIC was defined as the lowest concentration preventing visible growth but resuming growth upon re-plating onto CAP-free MRS agar. For minimum bactericidal concentration (MBC), 100 µL aliquots from wells with CAP concentrations above MIC were plated on CAP-free MRS agar after appropriate dilution. Following incubation at 30°C for 24 h, the lowest concentration yielding no colonies was defined as the MBC (14).

### Structure analysis of RelBE

The prediction of type II TAs across the genome of *W. cibaria* CGMCC 1.19376 was conducted using the Toxin-Antitoxin Database. The primary and secondary structure of RelE and RelB were performed by ClustalX and Jalview, and their promoter and terminator sequences were predicted by Softberry. The three-dimensional structures of RelE and RelB were predicted by SWISS-MODEL based on homology modeling methods. The RelB_2_ dimer was simulated by HDOCK, and the docking model of RelE-RelB_2_-RelE tetramer was displayed with PyMOL (4).

### Transcription analysis of RelBE and ClpXP in *W. cibaria*

The *W. cibaria* CGMCC 1.19376 was cultured in MRS broth with 1/2 MIC (4 μg/mL) CAP at 25°C for 6 h. Total RNA was extracted using RNAzol RT (Merck, Darmstadt, Germany). Subsequently, the cDNA synthesis was conducted using the PrimeScript RT reagent Kit (TaKaRa, Beijing, China). Co-transcription of *relB* and *relE* was examined by PCR using cDNA, mRNA, and genomic DNA as templates with primers listed in Table S1. For analysis of the antitoxin-degrading protease ClpXP, RT-PCR was conducted using *clpX* and *clpP* primers (Table S1) and the One Step PrimeScript RT-PCR Kit (TaKaRa, Beijing, China).

### Construction of recombinant *E. coli* BL21 with RelBE

The *rel*B and *rel*E genes from *W. cibaria* CGMCC 1.19376 were respectively ligated to plasmid pET28a and pBAD43, which contain a tightly tunable araBAD promoter that enables graded, low-level induction, to generate recombinant pET28a::*rel*B and pBAD43::*rel*E plasmids. Then, the two recombinant plasmids were co-transformed into *E. coli* BL21 (DE3) to obtain a recombinant *E. coli* PR. The expression of RelB and RelE was induced individually by 1 mM isopropyl β-D-1-thiogalactopyranoside (IPTG) and 0.1% L-arabinose, respectively. Growth curves were recorded to assess cytotoxic effects (4).

### Morphological analysis of persister cells

The persistent cells of *W. cibaria* CGMCC 1.19376 were obtained by the method of biphasic bactericidal curve (15). Briefly, *W. cibaria* CGMCC 1.19376 was cultured in MRS broth to about 1 × 10^7^ CFU/mL, then 4 μg/mL CAP was added to MRS and induced the expression of RelBE for 1–7 h. Subsequently, 0.5 mL cultures were incubated in 10 mL MRS broth containing MBC (512 μg/mL) CAP at 25°C for 24 h. The 1 mL incubation was collected via centrifugation at 4,000 rpm for 5 min and subsequently washed twice with sterile PBS (pH 7.4). Following resuspension and serial 10-fold dilution in sterile PBS (pH 7.4), the diluted samples were inoculated onto CAP-free MRS agar plates and incubated for 24 h at 25°C to facilitate colony counting. The frequency of persistent cells was designated as the ratio of viable bacteria in the RelBE expression versus the untreated control group.

To verify the regulatory effect of RelBE on the morphology of persister cells, RelB and/or RelE were induced in *E. coli* PR using 1 mM IPTG and/or 0.1% L-arabinose for 1–7 h. When only RelE was induced, persistent cells were isolated from *E. coli* PR after treatment with MBC (32 μg/mL) CAP in LB broth. Similarly, when both RelB and RelE were co-induced, a normal frequency of persistent cells occurred as RelB counteracted the toxicity of RelE. The frequency of persistent cells in *E. coli* PR was determined following a similar designation as described above. The morphology of persister and normal cells was observed using the FEI Inspect F50 scanning electron microscope (Inspect F50; FEI, Hillsboro, American) following Cai et al. (9).

### Impact of RelBE on the anabolism of the cell wall and cell membrane

The *W. cibaria* CGMCC 1.19376 was cultured in MRS broth with 4 μg/mL CAP at 25°C for 3 h, and the *E. coli* PR was induced with 0.1% L-arabinose in LB broth for 1 h at 30°C. The treatment without CAP or IPTG was the control. Total RNAs were extracted, followed by synthesis of double-stranded cDNA using the Takara PrimeScript Double Strand cDNA Synthesis Kit (TaKaRa, Beijing, China). The quantitative real-time PCR (RT-qPCR) was used to detect the transcription of genes related to cell wall and cell membrane synthesis; the primers are provided in Table S2. In RT-qPCR, the 16S rRNA was used as the internal reference gene. The cell membrane integrity and membrane potential were assessed as described by Xiong et al. (16).

### Impact of RelBE on the biofilm formation and cell division

The *W. cibaria* CGMCC 1.19376 and *E. coli* PR were respectively inoculated into a 96-well plate containing 200 μL of liquid medium with 4 μg/mL CAP or 0.1% L-arabinose. The absorbance at 600 nm was measured at 8, 12, and 16 h. Subsequently, the wells were gently washed twice with sterile PBS (pH 7.4) to eliminate free-floating cells. The plate was air-dried, followed by the addition of 200 μL of crystal violet stain and incubation for 30 min. Then, the crystal violet was removed, and the wells were washed twice with sterile PBS (pH 7.4). Finally, 200 μL of 95% ethanol was added to solubilize the crystal violet bound to the biofilm, and the absorbance at 540 nm was measured (17). The biofilm formation ability was quantified using the ratio of OD_540 nm_ and OD_600 nm_. Total RNAs were extracted, and then cDNA synthesis was conducted. RT-qPCR was performed to assess expression of genes associated with biofilm formation, the Sec secretion pathway, and cell division in *W. cibaria* at 25°C for 3 h and in *E. coli* PR at 30°C for 1 h. The primers are provided in Table S3.

### Statistical analysis

All experiments were conducted in triplicate, and the data were presented as the mean ± standard deviation, with variations less than 10%. Statistical significance analysis was carried out using analysis of variance (ANOVA) with Tukey’s post hoc test in SPSS 20.0, and a *P*-value less than 0.05 was considered statistically significant. Genes with significant expression differences were defined as ≥1.0 log_2_ or ≤−1.0 log_2_ relative to untreated controls (18).

## RESULTS

### RelBE activation enhances the phenotypic heterogeneity

**Fig 1:**
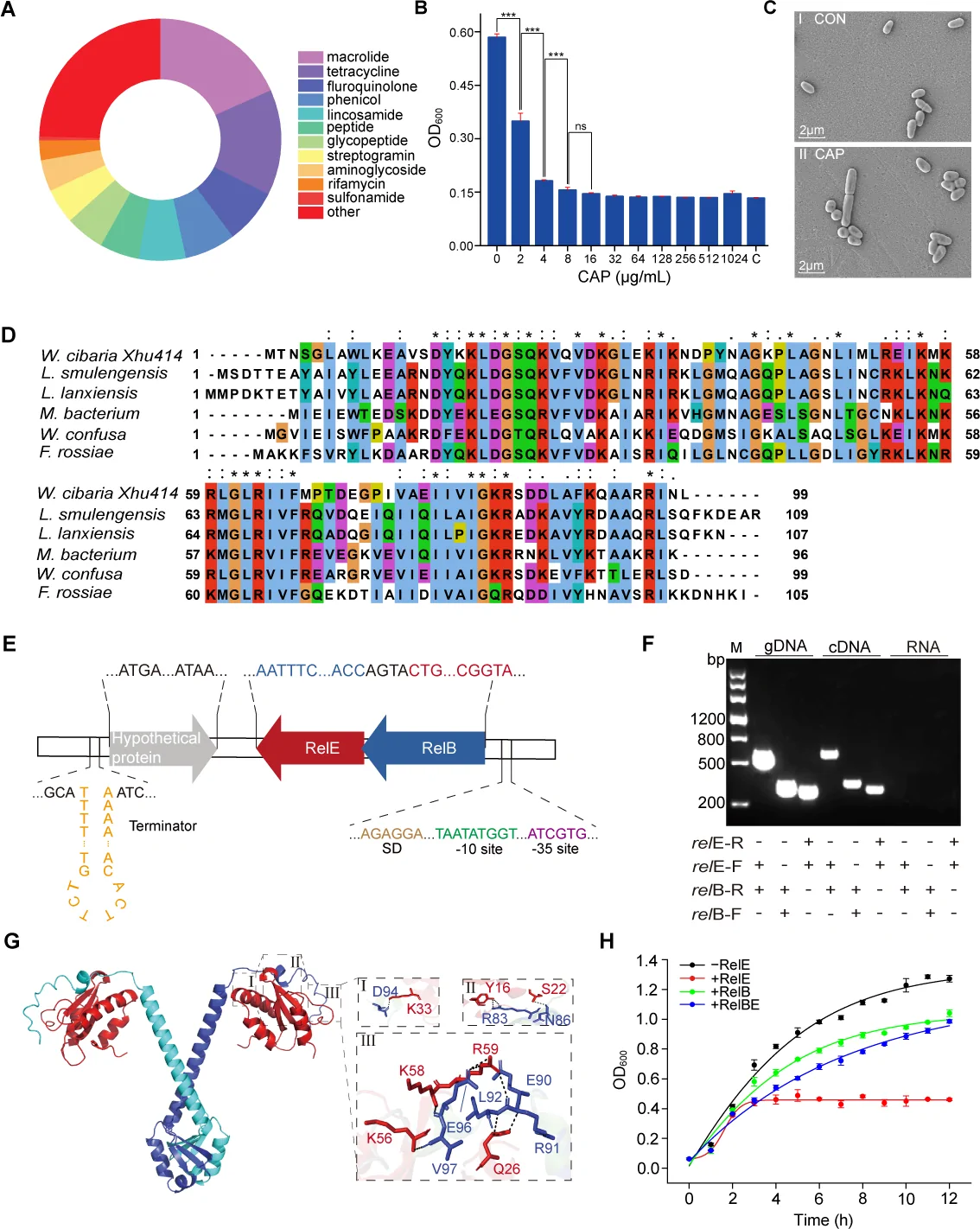
The structural and functional characteristics of RelBE in *W. cibaria*. (**A**) Antibiotic resistance genes in *W. cibaria*. (**B**) MIC determination of CAP against *W. cibaria*. (**C**) Morphological changes of *W. cibaria* after treatment with 4 μg/mL CAP. (**D**) Homologous alignment of RelE. Asterisk (*) denoted fully conserved amino acids, while colons (:) and periods (.) signified less conserved ones. (**E**) Operon structure of *rel*BE in *W. cibaria*. (**F**) Validation of *rel*BE co-transcription with gDNA-PCR, mRNA-RT-PCR, and mRNA-PCR. The three lanes in each group were *rel*BE, *rel*B, and *rel*E, respectively. (**G**) The silico-generated structure of RelE-RelB_2_-RelE tetramer. RelE was portrayed in red, and RelB was shown in blue and green, respectively. (**H**) Growth curve of *E. coli* PR with or without the expression of RelE and RelB.

Bacteria evade antibiotic stress through multiple mechanisms, including enzymatic inactivation, target protection, multidrug efflux, horizontal gene transfer, and adaptive mutations (19). Although the genome of *W. cibaria* CGMCC 1.19376 encodes several antibiotic resistance genes, no determinants specifically associated with CAP resistance were identified (Fig. 1A; Fig. S1). Nevertheless, this strain exhibited CAP tolerance to exceed the resistance threshold MIC (8 μg/mL) (Fig. 1B), consistent with resistance thresholds of the closely related genus *Leuconostoc* (20). Interestingly, at a subinhibitory CAP concentration (4 μg/mL), most cells retained a normal short-rod morphology (0.8–1.4 μm), whereas a distinct subpopulation displayed pronounced elongation (>4 µm; Fig. 1C), revealing marked phenotypic heterogeneity under CAP stress. These observations suggested that *W. cibaria* engaged the endogenous physiological programs in response to CAP, rather than relying solely on canonical resistance determinants.

Type II TA systems have been implicated in stress responses and growth modulation in specific contexts (21). Five putative type II TA operons were identified in the *W. cibaria* chromosome (Fig. S2). Upon exposure to 4 μg/mL CAP, all five operons exhibited upward transcriptional trends, with three significantly differentially expressed TAs (Fig. S3). Notably, the RelBE module displayed the strongest response, with *relE* and *relB* increasing by 2.56 and 2.87 log₂ fold, respectively. The RelE from *W. cibaria* CGMCC 1.19376 shares moderate sequence identity with homologs at 44%–50% (Fig. 1D), whereas RelB is less conserved (Fig. S4). The *relE* open reading frame (300 bp) lies downstream of *relB* with a four-base overlap (5′-ATGA-3′; Fig. 1E), and both genes are co-transcribed as a typical type II TA operon (Fig. 1F). Homology modeling indicates that RelE comprises four α-helices and four β-sheets, consistent with the *E. coli* RelE monomer (PDB 4FXI; Fig. S5), while RelB contains four α-helices and three β-sheets (PDB 3G5O). In solution, RelB forms a dimer and RelBE assembles as a RelE–RelB₂–RelE heterotetramer (Fig. 1G) (22).

In *E. coli* PR, controlled expression of RelE (15 kDa) markedly inhibited growth, whereas co-induction of RelB (16 kDa) restored normal proliferation (Fig. 1H; Fig. S6), confirming the canonical antagonistic relationship between the toxin and antitoxin. Together, these results indicated that RelBE was a CAP-responsive TA module in *W. cibaria* and that RelE activity was sufficient to impose growth arrest and phenotypic heterogeneity under defined experimental conditions. Rather than establishing a universal role in antibiotic tolerance, these data positioned the RelBE as a stress-responsive regulator whose activation under CAP coincided with growth modulation and the emergence of elongated subpopulations.

### RelE mediates the morphological remodeling of persister cells

**Fig 2:**
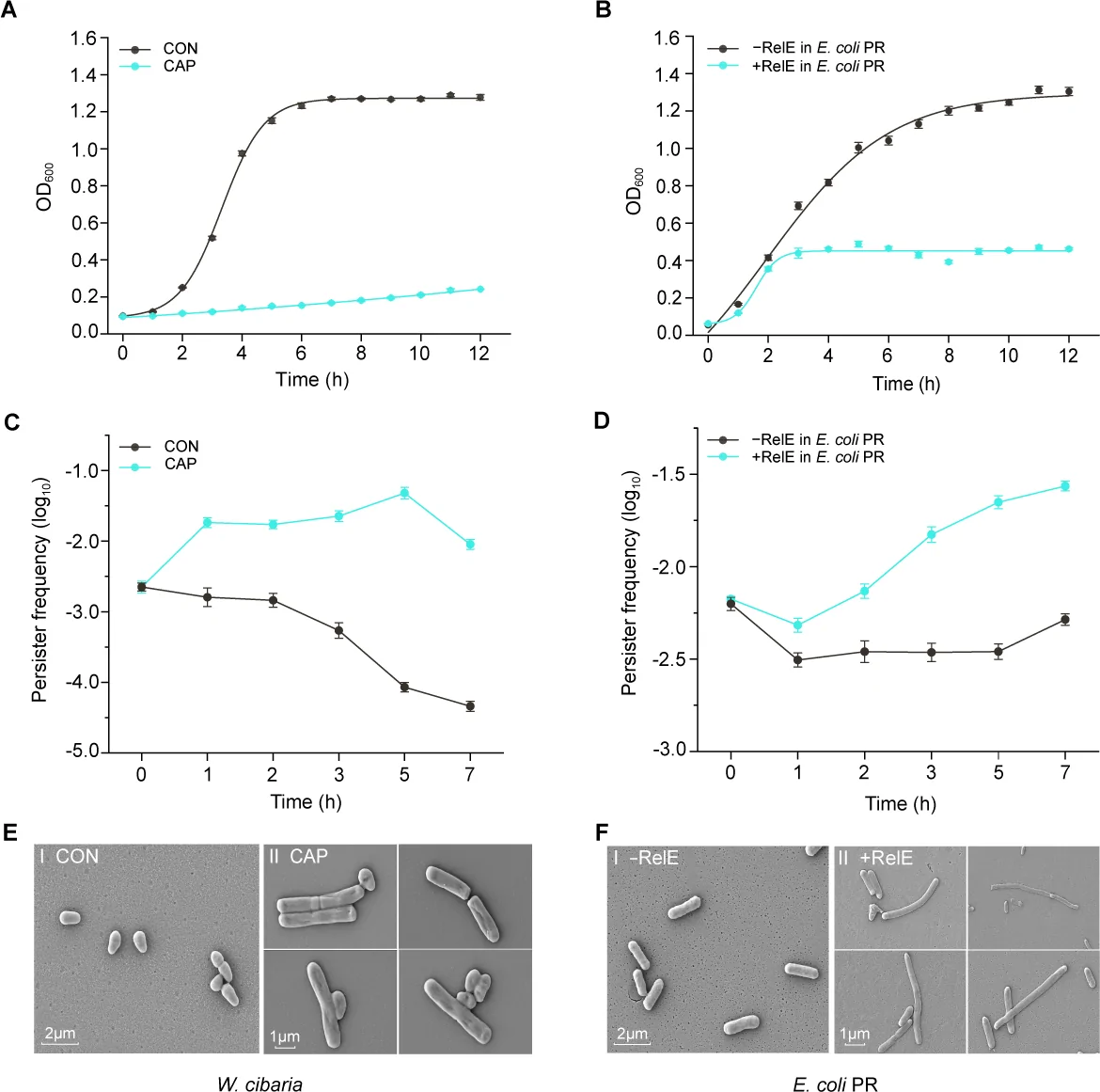
The effect of RelE on the cellular growth, persister formation frequency, and morphology of *W. cibaria* and *E. coli* PR. (**A**) Effect of RelE induction by 4 μg/mL CAP on the growth of *W. cibaria*. (**B**) Effect of RelE on the growth of *E. coli* PR. (**C**) Effect of RelE induced by 4 μg/mL CAP on the persister frequency of *W. cibaria*. (**D**) Effect of RelE on the persister frequency of *E. coli* PR. (**E**) Effect of RelE induced by 4 μg/mL CAP on the cellular morphology of *W. cibaria*. (**F**) Effect of RelE on the cellular morphology of *E. coli* PR.

Type II TA systems have been implicated in stress-responsive growth modulation in specific contexts, often through the transient accumulation of free toxin. Upon exposure of *W. cibaria* CGMCC 1.19376 to 4 μg/mL CAP, cellular growth was markedly slowed (Fig. 2A), concomitant with induction of both the RelBE module and the ClpXP protease, whose transcripts increased by 2.56–2.87 and 4.32–4.86 log₂ fold, respectively (Fig. S3 and S7). Given that ClpXP targets antitoxin RelB for degradation (23), this regulatory architecture is consistent with a transient rise in free RelE activity. In parallel, biphasic killing assays revealed a significant increase in post-treatment survival, with the apparent persister frequency shifting from −4.2 log_10_ to −1.3 log_10_ at a 5 h treatment (Fig. 2C). This change does reach the ≥2 log_10_ threshold commonly used to operationally define persister, indicating RelE-associated alterations in survival dynamics under CAP stress. Although in *E. coli* PR, the change in persister frequency did not meet this standard because of controlled induction of RelE, increasing only from −2.45 log_10_ to −1.55 log_10_, the RelE induction still resulted in a significant change (Fig. 2B and D).

Morphological remodeling is not an obligatory feature of persister-like states; however, when present, it frequently manifests as elongation or filamentation (4). Scanning electron microscopy revealed that CAP-induced activation of RelE in *W. cibaria* was consistently accompanied by pronounced cell elongation in a subpopulation (Fig. 2E). Cell length increased from an average of ~1.1 µm to >4.0 µm, with some cells reaching 5.78 μm. This phenotype was recapitulated in *E. coli* PR, where controlled RelE induction produced dramatic filamentation, with cell length increasing from ~2.1 µm to as much as 18.9 μm (Fig. 2F). These results demonstrated that RelE activity was sufficient to drive significant elongation under defined experimental conditions, implying a robust and reproducible association between RelE activation, growth arrest, and morphological remodeling, which provided a phenotypic anchor for subsequent mechanistic analyses of how toxin-mediated mRNA cleavage reshapes cellular architecture.

### RelE remodels the peptidoglycan anabolism of the cell wall

**Fig 3:**
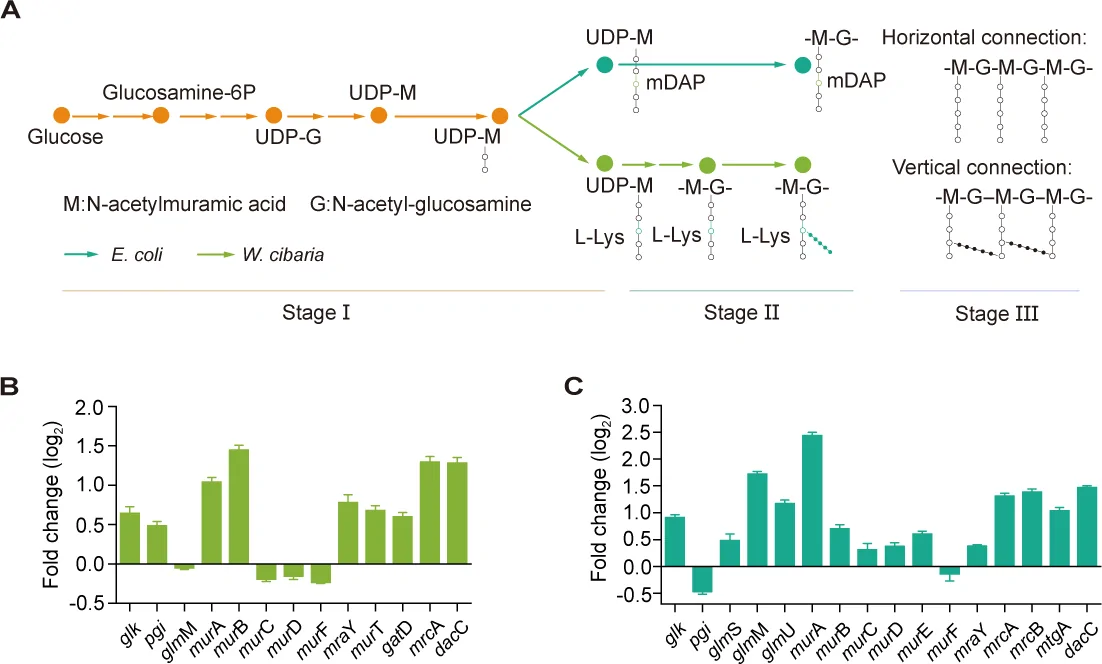
The effect of RelE on gene transcription in the cell wall synthesis pathway of *W. cibaria* and *E. coli* PR. (**A**) Schematic illustration of peptidoglycan biosynthesis pathways. The pathway specific to *W. cibaria* was illustrated in grass green (lower branch), whereas the pathway for *E. coli* PR was depicted in peacock green (upper branch). The common steps were highlighted in orange. (**B and C**) Effect of RelE on transcription of genes associated with cell wall synthesis in *W. cibaria* and *E. coli* PR, respectively.

The bacterial cell wall is a rigid structure essential for maintaining cell viability and determining cellular shape (24). In gram-positive bacteria, including lactic acid bacteria, the wall is primarily composed of peptidoglycan, formed by alternating N-acetylglucosamine (GlcNAc) and N-acetylmuramic acid (MurNAc) linked by β−1,4-glycosidic bonds (25). Short peptide stems (L-Ala–D-Glu–L-Lys–D-Ala–D-Ala) are attached to MurNAc residues and cross-linked via L-Lys bridges to generate a reticular scaffold. In contrast, gram-negative bacteria such as *E. coli* lack these peptide bridges and instead incorporate meso-diaminopimelic acid in the stem peptide. Peptidoglycan biosynthesis proceeds through three coordinated stages: synthesis of UDP-linked precursors (“Park” nucleotides), formation of peptidoglycan monomers, and polymerization and cross-linking of these monomers (Fig. 3A).

Under CAP exposure in *W. cibaria* CGMCC 1.19376, genes involved in the early precursor stage exhibited modest but coordinated changes. *glk* and *pgi* showed slight upregulation (0 < log₂ fold < 1), whereas *glm*M was mildly downregulated (0 > log₂ fold > −1; Fig. 3B). In *E. coli* PR following controlled RelE induction, *glk* was slightly upregulated, *gpi* slightly downregulated, and *glm*M and *glm*U were significantly upregulated by 1.78 and 1.26 log₂ fold, respectively (Fig. 3C). The initiating enzyme MurA, which catalyzes formation of UDP-GlcNAc enolpyruvate and represents a key entry point into peptidoglycan synthesis (26), was significantly induced in both systems with 1.12 log₂ fold in *W. cibaria* and 2.44 log₂ fold in *E. coli* PR. Other genes in this stage displayed only minor changes (−0.31 < log₂ fold < 0.68), with the exception of *mur*B, which was significantly upregulated by 1.46 log₂ fold in *W. cibaria* (Fig. 3B and C). At the level of stem peptide and bridge formation, *mur*T and *gat*D, which participate in peptide bridge synthesis in gram-positive bacteria, were modestly upregulated in *W. cibaria* (0.71 and 0.64 log₂ fold, respectively). In the final stage of polymerization, genes encoding enzymes mediating transglycosylation and transpeptidation, including *mrc*A and *dac*C, exhibited significant upregulation in both bacteria. In *W. cibaria*, *mrc*A and *dac*C increased by 1.24 log₂ fold each, while in *E. coli* PR, they rose by 1.36 and 1.45 log₂ fold, respectively (Fig. 3B and C). Collectively, these data indicated that RelE activation under CAP stress was accompanied by coordinated transcriptional remodeling across multiple steps of the peptidoglycan biosynthetic pathway, offering an explanation for the pronounced elongation phenotype observed under stress.

### RelE remodels the lipid anabolism of cell membrane

**Fig 4:**
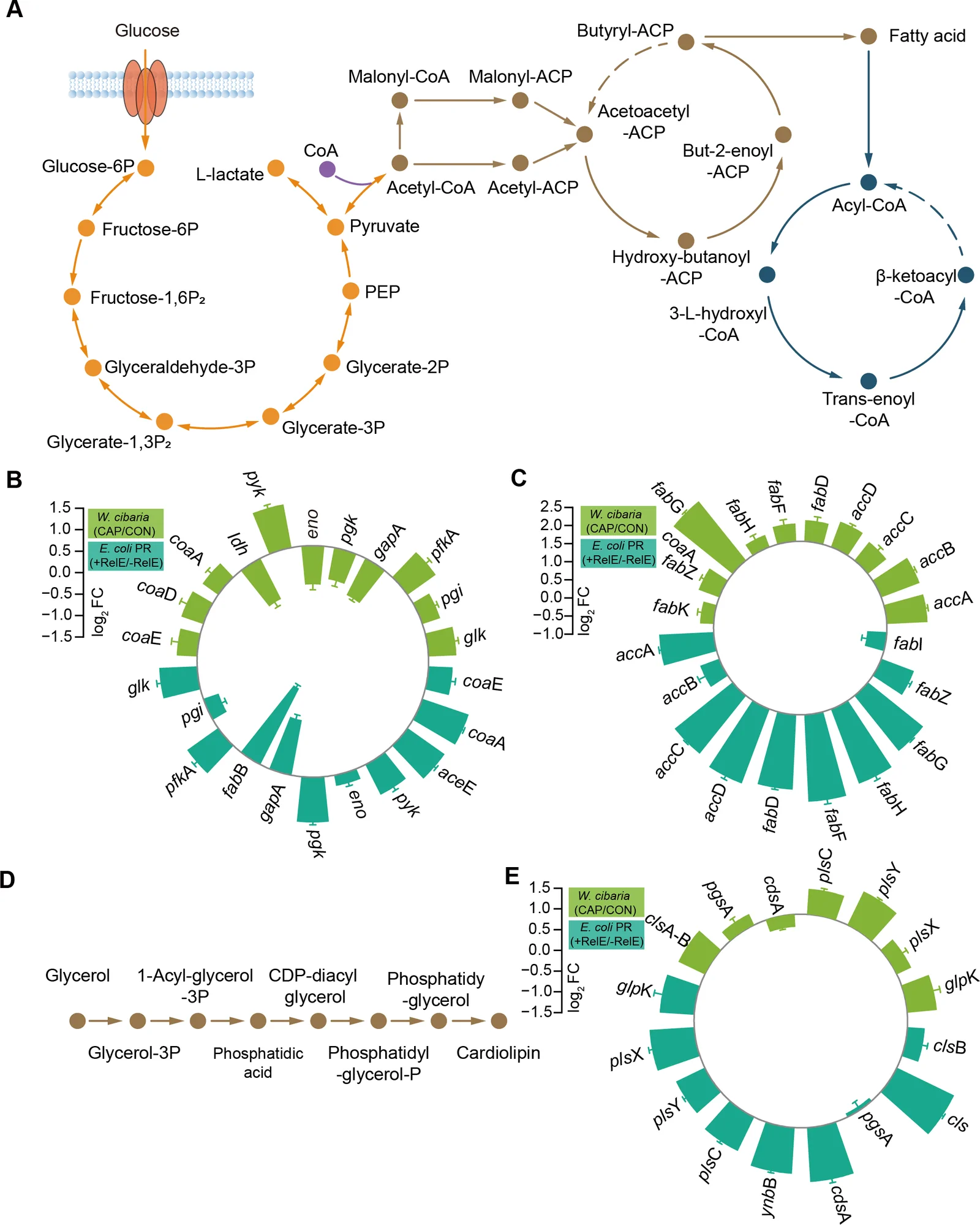
The effect of RelE on gene transcription in the cell membrane synthesis pathway of *W. cibaria* and *E. coli* PR. (**A**) Schematic illustration of fatty acid synthesis and metabolism pathways. Glycolytic processes were indicated by yellow lines, fatty acid biosynthesis by brown lines, fatty acid β-oxidation by dark green lines, and coenzyme A synthesis by purple lines. (**B**) Effect of RelE on transcription of genes in the glycolysis pathway in *W. cibaria* and *E. coli* PR. (**C**) Effect of RelE on transcription of genes in the fatty acid synthesis pathway in *W. cibaria* and *E. coli* PR. (**D**) Schematic illustration of cardiolipin and phosphatidylglycerol synthesis. (**E**) Effect of RelE on transcription of genes in cardiolipin and phosphatidylglycerol synthesis pathway in *W. cibaria* and *E. coli* PR.

The biosynthesis of membrane fatty acids and phospholipids is a central determinant for regulating cell morphology and size during bacterial proliferation. This process is tightly coupled to central carbon metabolism, as glycolysis (EMP pathway) supplies pyruvate, the key precursor for lipid biosynthesis. In lactic acid bacteria, pyruvate is predominantly converted into lactate via lactate dehydrogenase (Idh), but it can also be redirected toward acetyl-CoA production through pyruvate dehydrogenase (AceE), thereby feeding multiple anabolic pathways (Fig. 4A). Upon RelE activation, core glycolytic genes (*glk*, *pfk*A, and *pyk*) were upregulated in both *W. cibaria* CGMCC 1.19376 (0.73, 0.71, and 1.01 log₂ fold, respectively) and *E. coli* PR (0.88, 0.87, and 0.78 log₂ fold, respectively; Fig. 4B). In *W. cibaria*, however, *idh* was downregulated by −0.58 log₂ fold, indicating suppression of lactate production and a redirection of carbon flux. Consistently, genes involved in acetyl-CoA biosynthesis (*coa*A, *coa*D, and *coa*E) were upregulated, suggesting enhanced channeling of pyruvate toward lipid precursor pools.

Acetyl-CoA is converted into malonyl-CoA by acetyl-CoA carboxylase, a rate-limiting step in fatty acid biosynthesis (Fig. 4A). Following RelE activation, these genes were transcriptionally induced in both organisms. In *W. cibaria*, *acc*A and *acc*B increased by 1.18 and 1.16 log₂ fold, respectively, whereas in *E. coli* PR, *acc*A, *acc*C, and *acc*D rose by 1.45, 1.83, and 1.91 log₂ fold (Fig. 4C). Downstream enzymes, such as CoA-ACP transferase (*fab*D), ACP condensing enzyme (*fab*F and *fab*H), reductase I (*fab*G), dehydratase (*fab*Z), and reductase II (*fab*K, *fab*I), responsible for chain elongation and processing of acyl-ACP intermediates, were likewise upregulated in both bacteria, with the exception of *fabI*, which was reduced in *E. coli* PR (Fig. 4C). These coordinated changes indicated a broad reprogramming of fatty acid biosynthesis under RelE activity.

**Fig 5:**
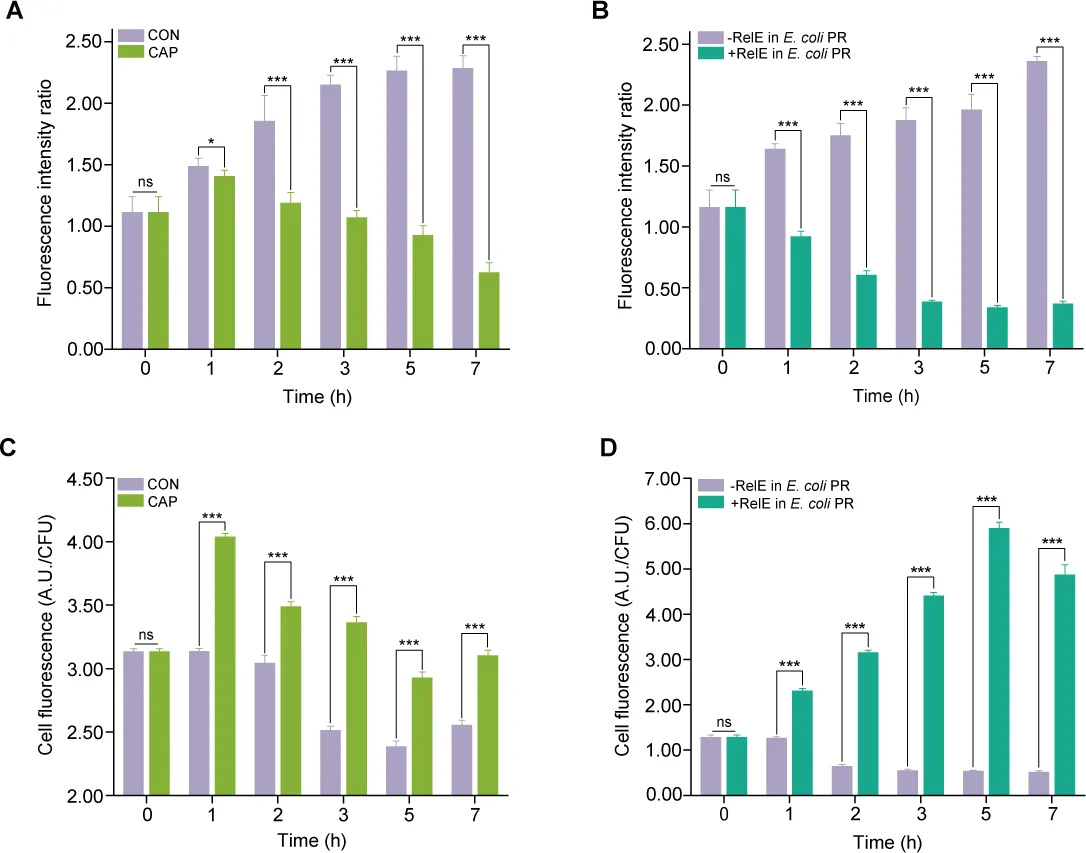
The effect of RelE on the cell membrane integrity and potential of *W. cibaria* and *E. coli* PR. (**A and B**) Effect of RelE on cell membrane integrity of *W. cibaria* and *E. coli* PR. (**C and D**) Effect of RelE on cell membrane potential of *W. cibaria* and *E. coli* PR. Dates were evaluated using Tukey’s post hoc test in conjunction with ANOVA. The asterisk (*) represented a significant level: *P < 0.05, ***P < 0.001, and ns *P* > 0.05.

Glycerophospholipids constitute the principal structural components of bacterial membranes. Phosphatidic acid, derived from glycerol-3-phosphate, serves as the central intermediate for the synthesis of phosphatidylglycerol, cardiolipin, and phosphatidylethanolamine (Fig. 4D). Focusing on phosphatidylglycerol and cardiolipin as representative glycerophospholipids, it was observed that nearly all genes involved in their biosynthetic pathways were upregulated upon RelE activation, with the exception of *cds*A in *W. cibaria* and *pgs*A in *E. coli* PR (Fig. 4E). This pattern suggested enhanced membrane lipid turnover or remodeling rather than a simple global suppression of anabolism. Disruption of membrane lipid homeostasis is expected to affect envelope integrity and electrochemical gradients. Consistent with this, CAP-induced RelE activation in *W. cibaria* resulted in a progressive decline of the SYBR Green I/PI ratio, reaching a 3.67-fold reduction relative to controls after 7 h (Fig. 5A). A comparable effect was observed in *E. coli* PR, where the ratio decreased by 6.52-fold following RelE induction (Fig. 5B), indicating compromised membrane integrity. Moreover, DiBAC₄(3) fluorescence, which increased upon membrane depolarization, rose markedly in both bacteria (Fig. 5C and D), revealing disruption of normal membrane potential. The observed reprogramming, which was accompanied by RelE activation, extensive transcriptional remodeling of lipid metabolic pathways, and functional impairment of membrane integrity and polarization, provided a mechanistic framework to explain how RelE-mediated mRNA cleavage could contribute to the morphological and biophysical changes observed under antibiotic stress.

### RelE promotes cell aggregation and biofilm formation

**Fig 6:**
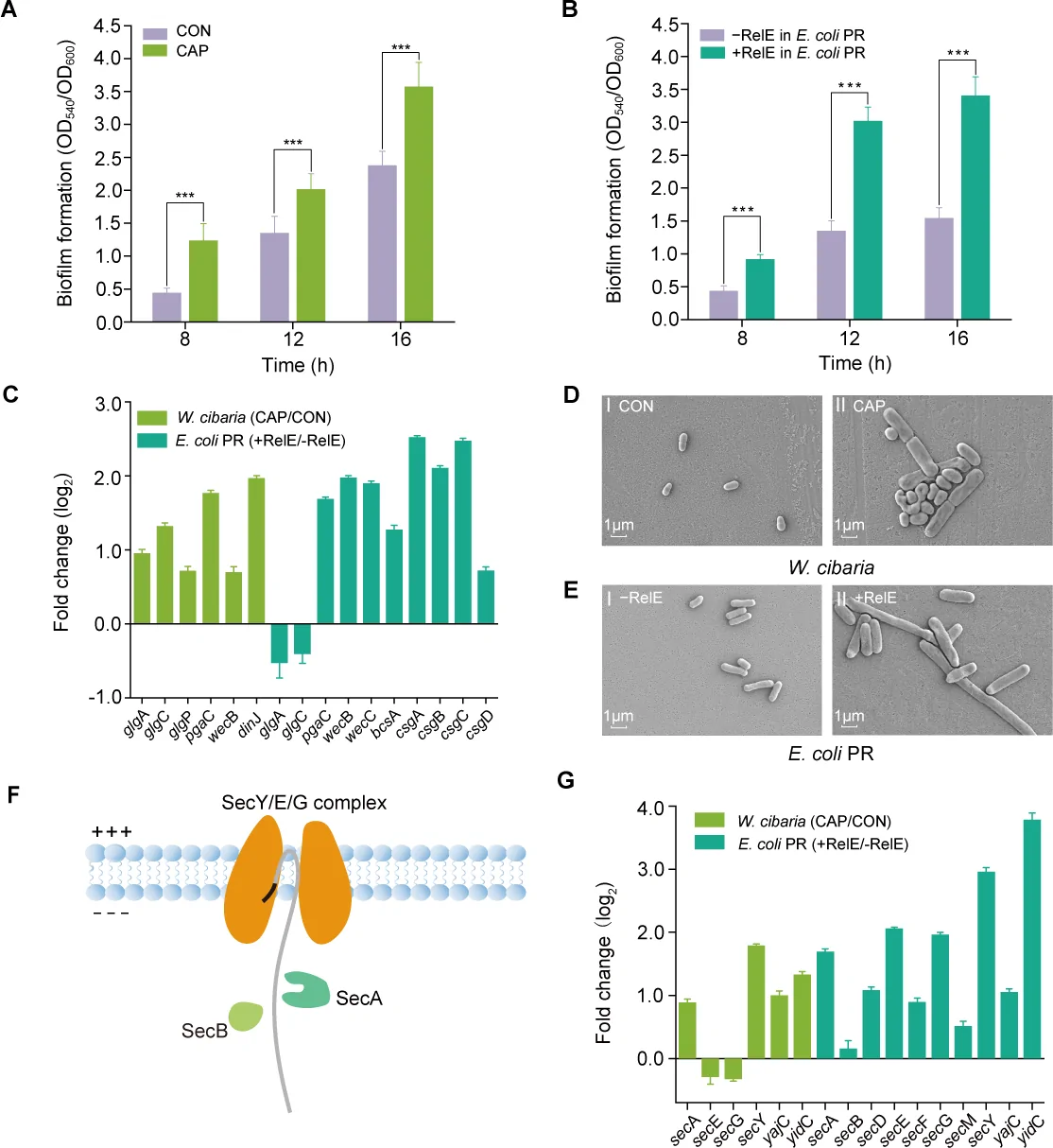
The effect of RelE on the biofilm formation of *W. cibaria* and *E. coli* PR. (**A and B**) Effect of RelE on the biofilm formation of *W. cibaria* and *E. coli* PR. (**C**) Effect of RelE on transcription of genes in biofilm formation in *W. cibaria* and *E. coli* PR. (**D and E**) Cellular aggregation of *W. cibaria* and *E. coli* PR with and without inducing RelE. (**F**) Schematic illustration of secretion pathway for Sec proteins. (**G**) Effect of RelE on transcription of genes in Sec protein secretion pathway in *W. cibaria* and *E. coli* PR. Dates were evaluated using Tukey’s post hoc test in conjunction with ANOVA. The asterisk (*) represented a significant level: ***P < 0.001.

TA systems have increasingly been implicated in biofilm-associated phenotypes, and cellular filamentation has been shown to facilitate surface attachment and biofilm formation (3, 27). Upon RelE activation, both *W. cibaria* CGMCC 1.19376 and *E. coli* PR exhibited a marked increase in biofilm biomass, rising by 1.51- to 2.91-fold and 1.15- to 2.21-fold, respectively (Fig. 6A and B). Consistent with this phenotype, transcriptional profiling revealed coordinated upregulation of biofilm-associated genes. In *W. cibaria*, the *glg*A, *glg*C, *pag*C, and *din*J were upregulated by more than 1.00 log₂ fold, whereas in *E. coli* PR, the *pag*C, *wec*B, *wec*C, and *bcs*A exhibited comparable upregulation (Fig. 6C). Notably, in *E. coli* PR, the curli fiber genes *csg*A, *csg*B, and *csg*C were strongly upregulated by 2.52, 2.10, and 2.47 log₂ fold, respectively. Curli fibers possess high adhesive capacity and self-assembly properties and constitute a core architectural element of bacterial biofilms (28). In parallel, RelE induction in both bacteria led to conspicuous cellular aggregation (Fig. 6D and E), providing a morphological correlate of the enhanced biofilm phenotype.

Initial attachment and matrix assembly during biofilm formation critically depend on the Sec-dependent protein secretion pathway (29). In gram-positive bacteria, which lack the canonical SecB chaperone, this pathway relies on functionally analogous components (30). The Sec machinery comprises the SecA, the membrane-embedded SecYEG translocon, and auxiliary factors including SecD, YajC, and YidC (Fig. 6F). In *W. cibaria* CGMCC 1.19376, RelE activation was accompanied by significant upregulation of *sec*Y, *yaj*C, and *yid*C, along with moderate induction of *sec*A, whereas *sec*E and *sec*G exhibited only minor decreases (Fig. 6G). In *E. coli* PR, all Sec pathway genes were upregulated, with *sec*B, *sec*D, *sec*G, *sec*Y, and *yid*C showing significant upregulation (Fig. 6G). These results indicated that RelE activation was accompanied by coordinated transcriptional changes in biofilm-associated pathways and in the Sec secretion machinery, coinciding with enhanced aggregation and biofilm formation to reshape the cell surface-associated behaviors under CAP stress.

### RelE mediates the repression of cell division programs

**Fig 7:**
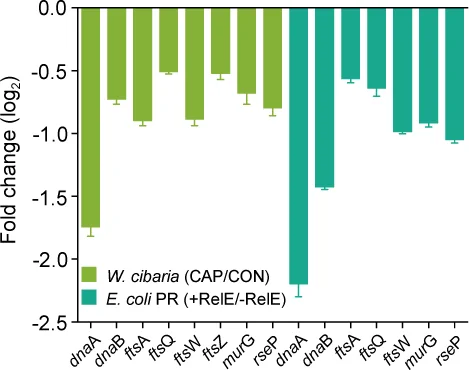
The effect of RelE on transcription of genes in cell division of *W. cibaria* and in *E. coli* PR.

Bacterial elongation is commonly linked to impaired septation and disruption of the cell division machinery (3). In *W. cibaria* CGMCC 1.19376, CAP-induced activation of RelE was accompanied by a coordinated downregulation of genes involved in cell division. Among these, the chromosome replication initiator *dna*A exhibited a pronounced downregulation with −1.73 log₂ fold, indicating suppression of early cell cycle events. In *E. coli* PR, this effect was even more pronounced, with significant downregulation observed in *dna*A, *dna*B, *fis*W, and *rse*P (Fig. 7). The stronger transcriptional repression in *E. coli* PR was consistent with the more dramatic filamentation observed in this host relative to *W. cibaria*. These data demonstrated a strong correlation between RelE activity and the inhibition of the division program, offering a mechanistic rationale for the observed elongation phenotype resulting from the suppression of division and the maintenance of a filamentous morphology.

## DISCUSSION

**Fig 8:**
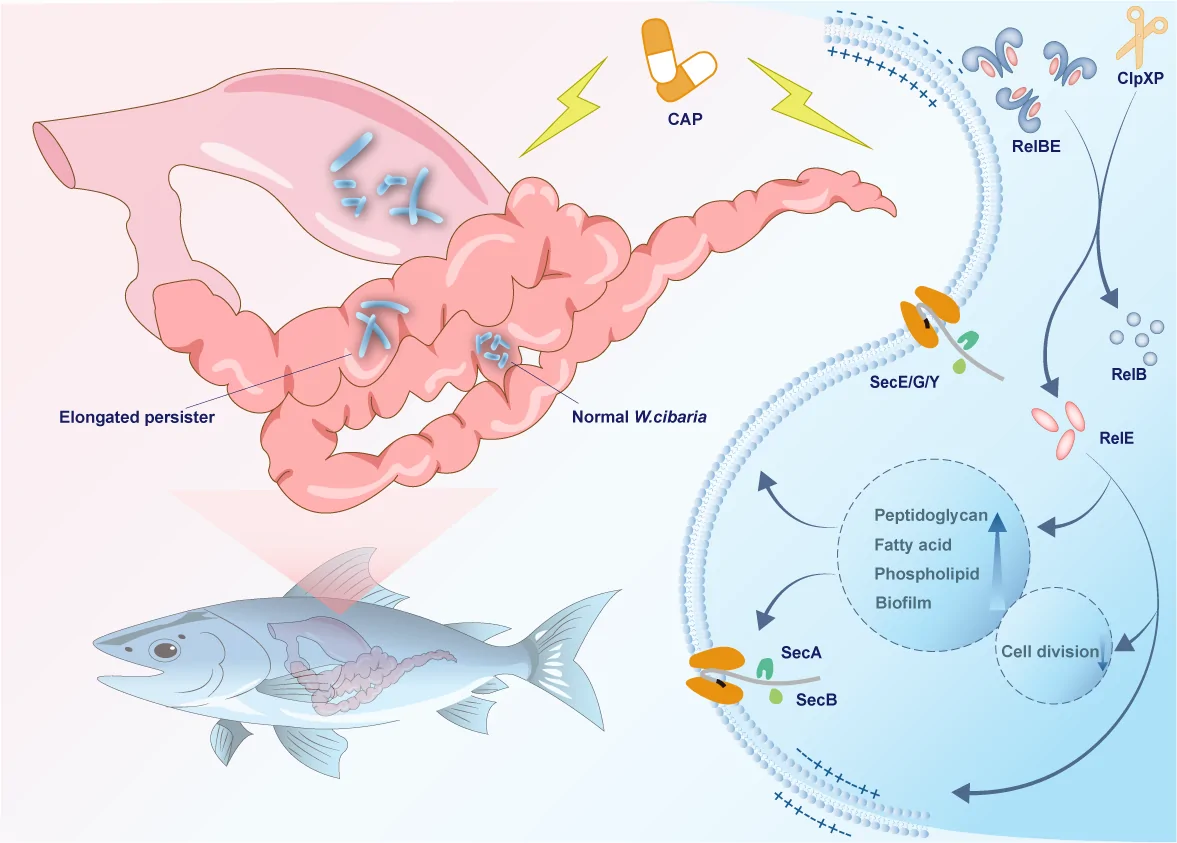
The schematic diagram illustrating the mechanism by which RelBE enables *W. cibaria* to evade residual CAP in the intestinal environment of fish.

It has largely remained mysterious how the molecular features and activities of TA modules are translated into biological function (5). The current study demonstrated that the RelBE TA module of *W. cibaria* CGMCC 1.19376 was robustly responsive to CAP stress and that controlled RelE activity was associated with growth arrest, phenotypic heterogeneity, and pronounced cell elongation. Rather than establishing a universal or deterministic role for RelBE in persister formation, our data delineated a mechanistic link between toxin activation and morphological remodeling under CAP stress. RelE, an mRNA endonuclease that preferentially cleaves ribosome-associated transcripts (31), reshapes cellular physiology in a manner that is sufficient to reproduce a persister state under defined experimental conditions. Through this mechanism, *W. cibaria* CGMCC 1.19376 can adeptly avoid CAP treatment and ensure its safe arrival in the intestine to exert probiotic functions (Fig. 8). In the absence of CAP, RelBE was maintained at low levels as a non-toxic RelE-RelB₂-RelE complex. Upon CAP exposure, induction of the ClpXP protease promotes antitoxin degradation, transiently increasing free RelE. This shift coincides with broad reprogramming of envelope biogenesis, suppression of cell cycle progression, and enhancement of surface-associated behaviors.

Cell shape is tightly coupled to the integrity and dynamics of the cell wall and membrane (32, 33). In gram-positive bacteria, several type II toxins, including Zeta and PezT, impair peptidoglycan synthesis through direct interference with UDP-linked precursors (34, 35). The toxin protein ng-ζ1 from the gram-negative *Neisseria gonorrhoeae* also exhibits a similar function to disrupt cell wall formation by interfering with peptidoglycan synthesis (36). Consistent with this paradigm, RelE activation in *W. cibaria* and *E. coli* PR was accompanied by coordinated transcriptional changes across multiple stages of peptidoglycan biosynthesis, encompassing precursor formation, stem peptide/bridge assembly, and polymerization. Specifically, it influenced the transcription of genes associated with UDP-N-acetylamino-aminoenolpyruvate, the cell wall peptide bridge, and the peptidoglycan monomer linkage (Fig. 3B and C). While we did not suggest that these genes were uniquely targeted by RelE, the observed pattern provided a mechanistic framework linking toxin activity to altered envelope biogenesis and impaired septation, offering an explanation for the elongation phenotype.

Membrane lipid homeostasis represents a second axis through which RelE reshapes cellular architecture. Previous work has shown that fatty acid synthesis rates directly modulate bacterial cell size (37). Here, RelE activation redirected carbon flux toward acetyl-CoA and malonyl-CoA production and induced the acetyl-CoA carboxylase complex, the rate-limiting step in fatty acid synthesis. Downstream *fab* genes and glycerophospholipid pathways were broadly upregulated, coinciding with compromised membrane integrity and depolarization. These findings were most parsimoniously interpreted as a compensatory response to envelope destabilization: RelE-mediated mRNA cleavage perturbed membrane homeostasis, and cells attempted to restore integrity by upregulating lipid biosynthesis. Such compensation, however, occurred in a context of growth arrest and division blockade, favoring elongation and filamentation.

Filamentation is a common outcome of impaired division, and several type II toxins restrain septation, producing elongated cells under stress (3, 38). In our study, RelE activation coincided with strong repression of replication and division genes, including *dna*A, *dna*B, and multiple *fts* components (Fig. 7). This transcriptional landscape was incompatible with normal cell cycle progression and provided a direct mechanistic basis for filamentation. The effect was more pronounced in *E. coli* PR than in *W. cibaria*, consistent with the greater degree of elongation observed in the heterologous host. RelE also reshaped community-level behaviors. Biofilm biomass increased in both bacteria, accompanied by the induction of biofilm-associated genes and the Sec secretion machinery. In *E. coli* PR, strong upregulation of curli fiber genes promoted aggregation and surface adhesion (39), further reinforcing the filamentous phenotype (Fig. 6). These changes link toxin-mediated growth modulation to surface-associated adaptations that are advantageous under stress.

Importantly, morphological remodeling is not an obligatory hallmark of persistence, and RelE should not be viewed as a universal persister generator. Our approach was hypothesis driven and pathway focused, aiming to define how toxin activity can impinge on cellular systems governing morphology. We therefore interpreted our results as establishing a robust association between RelE activation, envelope remodeling, division repression, and elongation under CAP stress, rather than as definitive proof of *in vivo* persister determination. Undoubtedly, genome-wide transcriptomics and genetic dissection of TA networks will be essential to place RelBE within the broader regulatory architecture of stress responses.

In summary, RelBE contributed to stress-induced morphological adaptation in *W. cibaria* by coupling RelE-mediated mRNA cleavage to coordinated reprogramming of envelope biogenesis, cell cycle control, and biofilm-associated pathways, culminating in cell elongation. These findings revealed a mechanistic route by which toxin activity reshapes cellular architecture under antibiotic stress and provide a framework for evaluating probiotic performance in antibiotic-impacted environments. Future work employing targeted knockouts of *relBE* and other TA modules, together with genome-wide profiling, will also be required to resolve their specific and collective roles in morphology and persistence.

## Data Availability

The genome sequence of *W. cibaria* CGMCC 1.19376 (Xhu414) is publicly available in NCBI under accession numbers PRJNA1269134 and GCA_050908175.1.

## SUPPLEMENTAL MATERIAL

Supplemental material spectrum.02518-25-s0001.docx Fig. S1 to S7; Tables S1–S3.

ASM does not own the copyrights to Supplemental Material that may be linked to, or accessed through, an article. The authors have granted ASM a non-exclusive, world-wide license to publish the Supplemental Material files. Please contact the corresponding author directly for reuse.
