# Supplementary material for: RelBE-mediated stress responses reshape cellular morphology of Weissella cibaria under chloramphenicol exposure
Source: Microbiol Spectr. 2026 May 18;14(7):e02518-25. doi: 10.1128/spectrum.02518-25 (PMC13340145; doi:10.1128/spectrum.02518-25)
Supplement: Supplemental material — Fig. S1 to S7; Tables S1–S3. [file spectrum.02518-25-s0001.docx]

**Supplementary Figures and Tables**


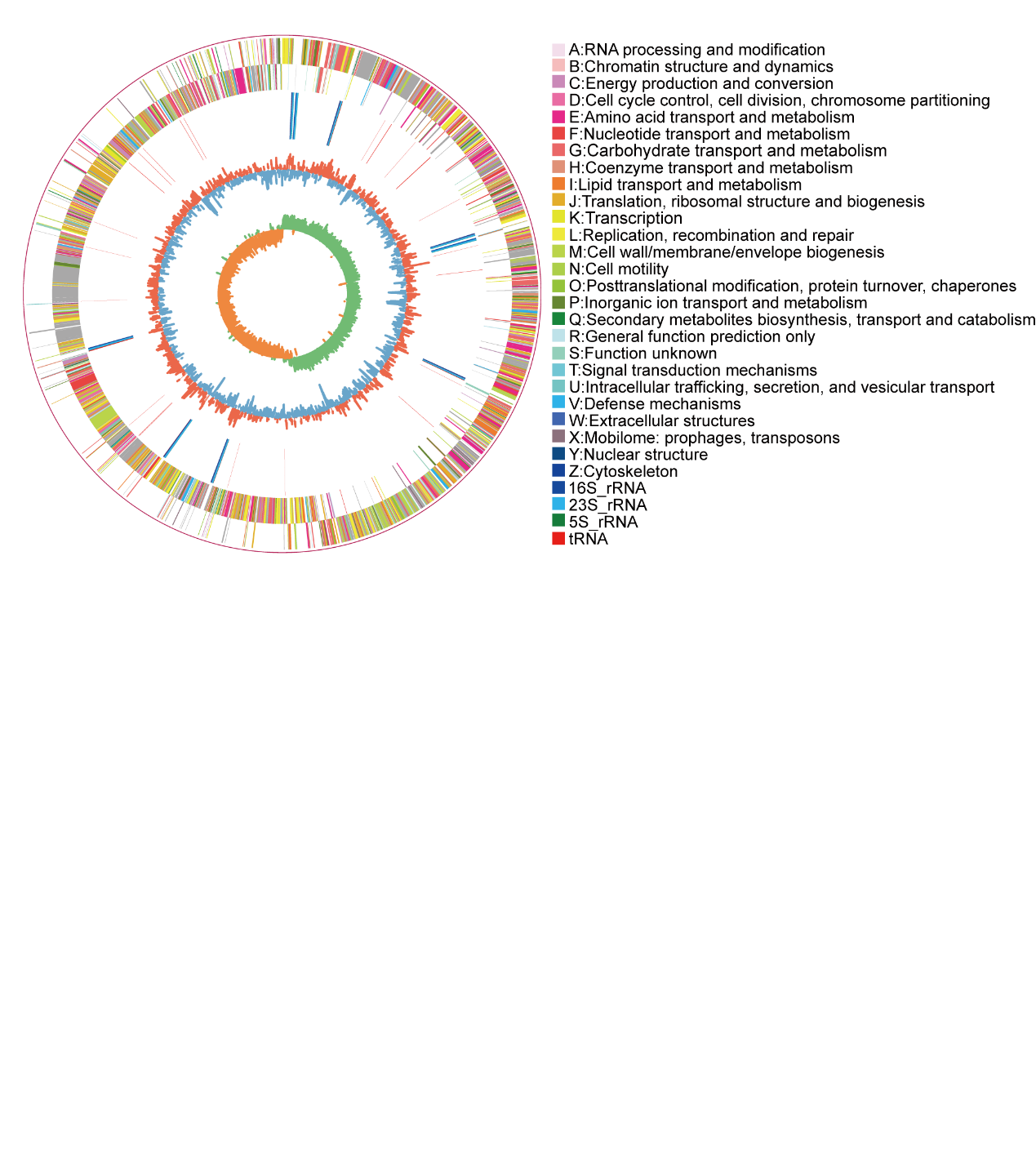


**FIG S1.** Circle map of the genome of *W. cibaria* CGMCC 1.19376. The outermost layer indicates the size of the genome. Different colors denote the various COG functions of the CDSs on the positive and negative chains represented by the second and third circles. The fourth loop are rRNA and tRNA, the fifth is GC content, and the innermost circle is GC-Skew.


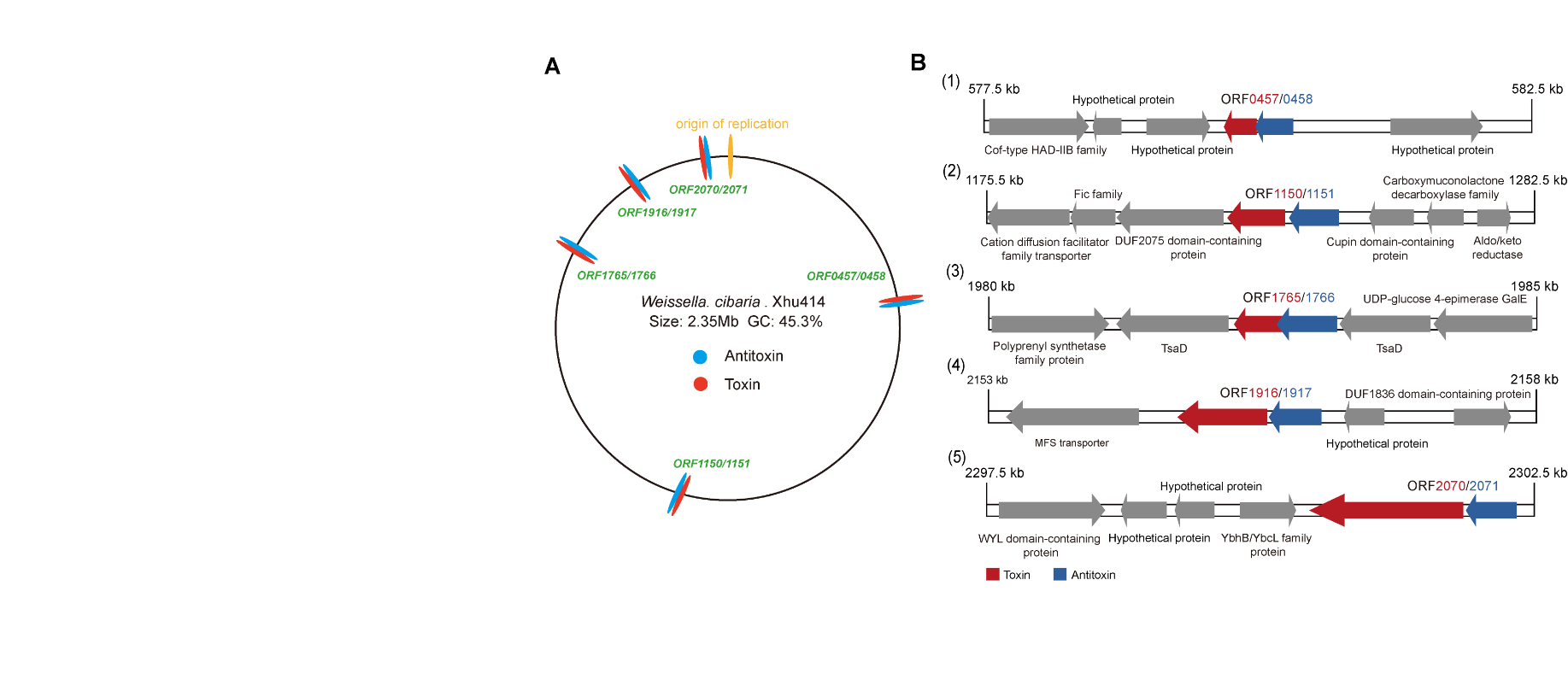


**FIG S2**. Type II TAs in genome of *W. cibaria* CGMCC 1.19376 (Xhu414). (A) Distribution of 5 type II TAs in the genome. (B) Operon structure of 5 type II TAs.


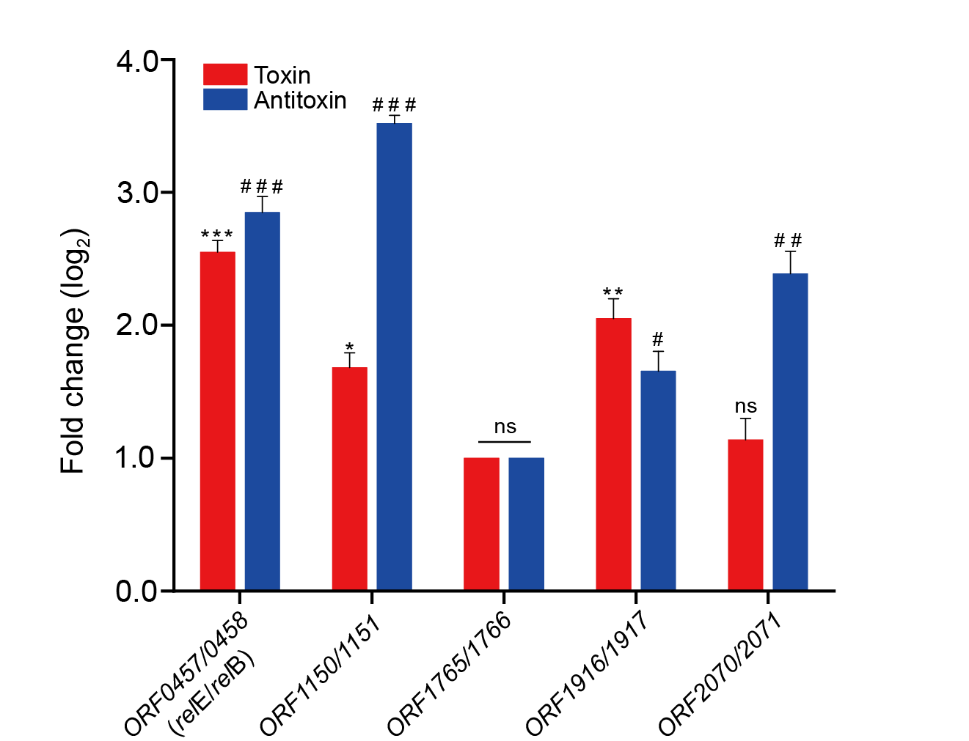


**FIG S3**. Fold change in the expression of type II TAs under 1/2 MIC CAP. The asterisk (*) or hashtag (#) represent significant level: */# *P* < 0.05, **/## *P* < 0.01, ***/### *P* < 0.001, ns P > 0.05.


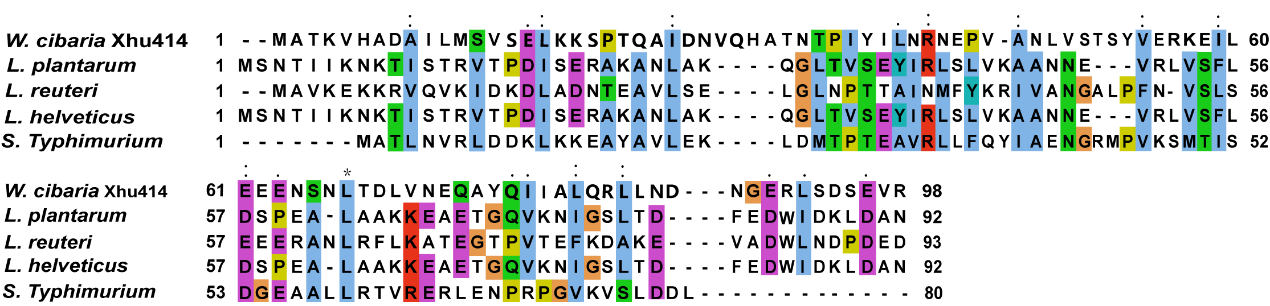


**Fig. S4**. Alignment of homologous RelB from bacteria.


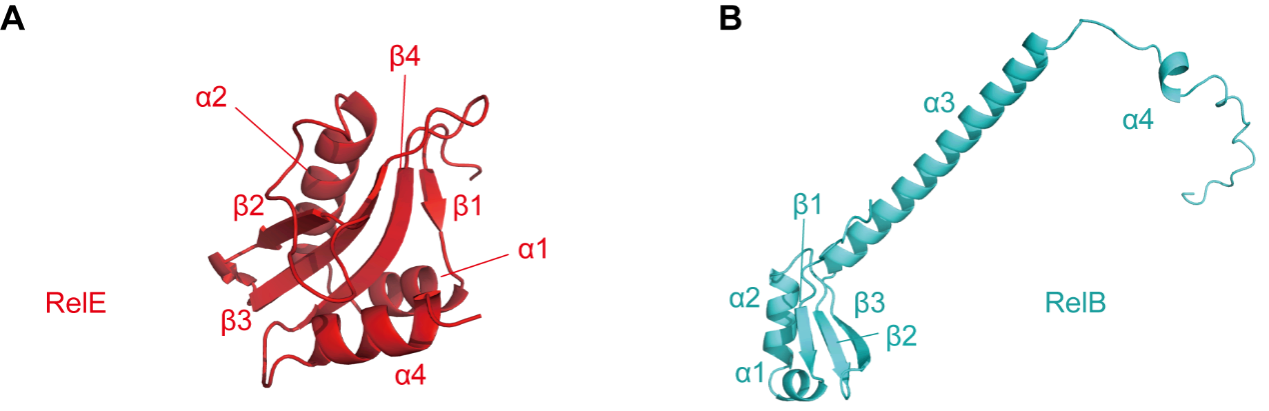


**FIG S5**. (A) Teriary sctructer model of RelE (A) and RelB monomer (B) form *E. coli* K-12.


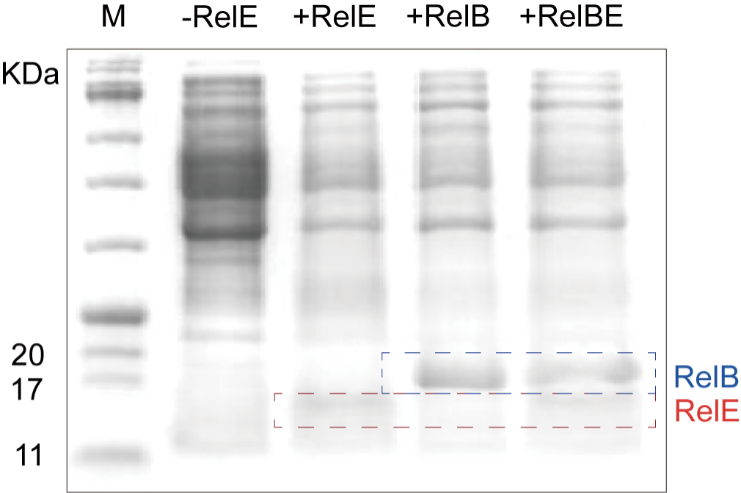


**FIG S6**. In vitro denatured PAGE separation of RelE (in red box) and RelB (in blue box) in *E. coli* PR.


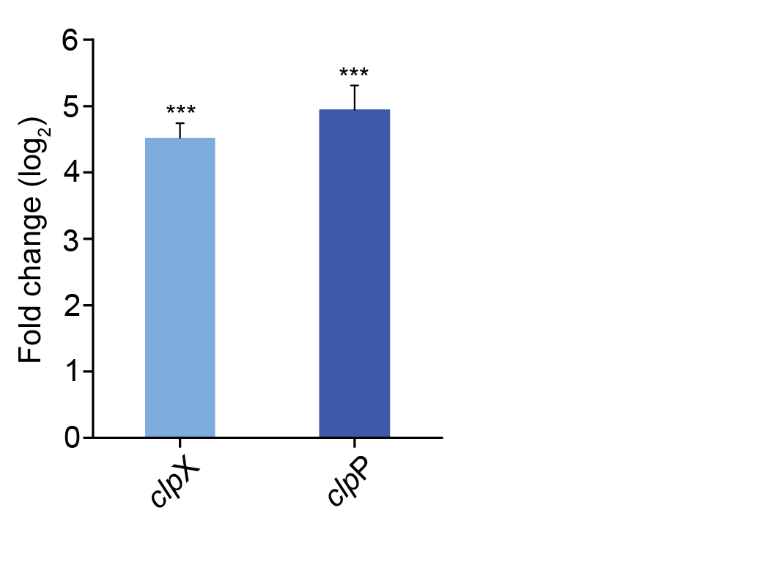


**FIG S7**. Expression of *clp*XP in *W. cibaria* under 1/2 MIC CAP

**TABLE S1** Primers of TAs and *clp*XP in *W. cibaria* CGMCC 1.19376

| Pimer | Sequence（5’ - 3’） | Bacterial strain |
| --- | --- | --- |
| *ORF0457*-F (*relE*) | CCAATAGTGGCCTAGCCTGG | *W. cibaria* |
| *ORF0457*-R (*relE*) | GTTACCAGCTAACGGCTTGC | *W. cibaria* |
| *ORF0458*-F (*relB*) | TAGATGGGCGTATTGGTGGC | *W. cibaria* |
| *ORF0458*-R (*relB*) | AAGTACACGCAGACGCCATT | *W. cibaria* |
| *ORF1150*-F | CAACTTCAAGCGCATCCTCG | *W. cibaria* |
| *ORF1150*-R | ACATGCGTGATTACCTGGCA | *W. cibaria* |
| *ORF1151*-F | GGCGTTCAATTGTTGCACCT | *W. cibaria* |
| *ORF1151*-R | TGTCGCGGGCAGTTAAGAAA | *W. cibaria* |
| *ORF1765*-F | CCAATTGGTTCGCCATCAGC | *W. cibaria* |
| *ORF1765*-R | CAATTGCCAAAGCGGCGTAT | *W. cibaria* |
| *ORF1766*-F | GCACTTCCAGTGACATCCGA | *W. cibaria* |
| *ORF1766*-R | TCCACGAGGTGCACATTACC | *W. cibaria* |
| *ORF1916*-F | AATTGCCGGTCACGTAGGTT | *W. cibaria* |
| *ORF1916*-R | TGTTCCGCGAGTACAAGCAT | *W. cibaria* |
| *ORF1917-*F | CCTGTCCCACCTGACGTAAC | *W. cibaria* |
| *ORF1917-*R | TTCATACGGAAGCAGAGGCG | *W. cibaria* |
| *ORF2070-*F | ATTGACATCACACGGCCCAT | *W. cibaria* |
| *ORF2070-*R | TTGTTAGCCGGTAGTGCCTG | *W. cibaria* |
| *ORF2071-*F | TGACGCCGGTTAAGTTCCTC | *W. cibaria* |
| *ORF2071-*R | GGCGGCGTGACAAAATTGAT | *W. cibaria* |
| *clpX*-F | TGCTTCAACTTGGGTTGGGT | *W. cibaria* |
| *clpX*-R | ATTGGCTGCCGACTTGATGA | *W. cibaria* |
| *clpP*-F | GACCAAACACCACCGTTTCG | *W. cibaria* |
| *clpP*-R | AAGCCAGCCATCCACTACAC | *W. cibaria* |
| *16S*-F | TCACCGGCAGTCTCACTAGA | All |
| *16S-*R | ACGCGAAGAACCTTACCAGG | All |

**TABLE S2** Primers of genes related to cell wall and membrane synthesis

| Pimer | Sequence（5’ - 3’） | Bacterial strain |
| --- | --- | --- |
| *glk-*F | GAGATGGTGCCTGGTGAGTC | *W. cibaria* |
| *glk-*R | CCGACGAACTGTGGTGATGA | *W. cibaria* |
| *pgi-*F | AGACGTTGGCTGATGCAGAA | *W. cibaria* |
| *pgi-*R | CAGCGATTGGCAACAAACCA | *W. cibaria* |
| *glmM-*F | TTGCCATTGATGCAGCCAAC | *W. cibaria* |
| *glmM-*R | TGATGTGGCCATCGTGACAA | *W. cibaria* |
| *murA-*F | GCGTGGCAATTGAAAACCCT | *W. cibaria* |
| *murA-*R | CATTAGGCCAGCAGTGACCA | *W. cibaria* |
| *murB-*F | TGTTACGCAAATTGCCCGTG | *W. cibaria* |
| *murB-*R | GCATTCATGAAGACCGCACC | *W. cibaria* |
| *murC-*F | TTTGGCCGTGATTGCTGTTG | *W. cibaria* |
| *murC-*R | GGGGCCAACTTGCTTTTCAG | *W. cibaria* |
| *murD-*F | TGCGGCTTCATGGGATCAAT | *W. cibaria* |
| *murD-*R | CTTTCGTAGCGTGTGCTTGG | *W. cibaria* |
| *murF-*F | CGGTATGGATCGTCCTGGTG | *W. cibaria* |
| *murF-*R | TGCGTTCGCGAGTTTTGAAG | *W. cibaria* |
| *mraY-*F | GCTTCGAAAAGGGTGGCTTG | *W. cibaria* |
| *mraY-*R | CACTGCCCAGAAGAACGAGT | *W. cibaria* |
| *murT-*F | AGGGCATTCGTTTGTTCCCT | *W. cibaria* |
| *murT-*R | CCTTCATATCGCCATCCGCT | *W. cibaria* |
| *gatD-*F | GCGGTTTCGAAAACCACCAA | *W. cibaria* |
| *gatD-*R | TACACAAGTCCTTCACCGCC | *W. cibaria* |
| *mrcA-*F | ATTGGACGATGCCGGATACC | *W. cibaria* |
| *mrcA-*R | GTTCACGTTGGACAGTTGGC | *W. cibaria* |
| *dacC-*F | AAGACGGCGGTCTCAATGTT | *W. cibaria* |
| *dacC-*R | GTGCCCCCATCAACGAAAAC | *W. cibaria* |
| *pfkA-*F | ATTTGTCGGGGGCAAAGCTA | *W. cibaria* |
| *pfkA-*R | ACCTTTACGGCGACGGATTT | *W. cibaria* |
| *gapA-*F | GCACGGTACTTTGCCAGTTG | *W. cibaria* |
| *gapA-*R | ACTTCAAGTCAGCCGCGTTA | *W. cibaria* |
| *pgk-*F | TGGCCTTTACGTTCGATGCT | *W. cibaria* |
| *pgk-*R | TTGTCGCCAGCTTCTTCGAT | *W. cibaria* |
| *eno-*F | AGCTGGTTACACGGCTATCG | *W. cibaria* |
| *eno-*R | TGATTTGACCCGCGTTCGTA | *W. cibaria* |
| *pyk-*F | ACGCCGTATTTGACGGAACT | *W. cibaria* |
| *pyk-*R | GATACGGGCCATCGTAGCAA | *W. cibaria* |
| *ldh-*F | TTGCTAAGCTGGCTGATGCT | *W. cibaria* |
| *ldh-*R | GCCCATATCATCACGTCGGT | *W. cibaria* |
| *coaA-*F | GCGTGATCGTGAAGAACGTG | *W. cibaria* |
| *coaA-*R | ACCATACTCCGCTTGCAACA | *W. cibaria* |
| *coaD-*F | TGCGTCGGGTATTGTTTCCA | *W. cibaria* |
| *coaD-*R | CTACGCCGATGACCACTTCA | *W. cibaria* |
| *coaE-*F | ACGTCTCATGGCACGCAATA | *W. cibaria* |
| *coaE-*R | TGCTAACACGGCATCCACTT | *W. cibaria* |
| *accA-*F | GTAAGTCCGCGGAAGACCAA | *W. cibaria* |
| *accA-*R | GCCCCCTTCCCCATAAACAA | *W. cibaria* |
| *accB-*F | CACTAACAATCACGGCCCCA | *W. cibaria* |
| *accB-*R | CCACAATAGTCCCGGCTACC | *W. cibaria* |
| *accC-*F | ATTGTTGCTGAAAGCCGCTG | *W. cibaria* |
| *accC-*R | CACGCATCACTTGCATCTCG | *W. cibaria* |
| *accD-*F | TGTGGCTATGGTTTCCGCTT | *W. cibaria* |
| *accD-*R | CGCCCAATTCCGTTTGTTGT | *W. cibaria* |
| *fabD-*F | CCAGGTAAGATGGTTGCCGT | *W. cibaria* |
| *fabD-*R | CGTGAGACACCTGCTTCAGT | *W. cibaria* |
| *fabF-*F | GGTTACGGGGCAAACTCTGA | *W. cibaria* |
| *fabF-*R | ATTTGCACCAGTTGCCGTTC | *W. cibaria* |
| *fabH-*F | CATCGTGACGACGTTTACGC | *W. cibaria* |
| *fabH-*R | AGACAAATCCTGAGCACGCA | *W. cibaria* |
| *fabG-*F | AGACAAATCCTGAGCACGCA | *W. cibaria* |
| *fabG-*R | GCCGCATTCATTGGCGTATT | *W. cibaria* |
| *fabZ-*F | GAGATGGTGCCTGGTGAGTC | *W. cibaria* |
| *fabZ-*R | CCGACGAACTGTGGTGATGA | *W. cibaria* |
| *fabK-*F | TTGCACAGCCACGGTATCTT | *W. cibaria* |
| *fabK-*R | CCGCCGCAATAACTGGAATG | *W. cibaria* |
| *glpK-*F | CTGAAGCTGGCAACCTGGTA | *W. cibaria* |
| *glpK-*R | TACTCGTGTGAACCTGTGGC | *W. cibaria* |
| *plsX-*F | GGCTGATGAGCCAGTGAAGT | *W. cibaria* |
| *plsX-*R | GAGAAAATGGCATCCGCGTC | *W. cibaria* |
| *plsY-*F | TGCCGTTTGGCTACTGGATT | *W. cibaria* |
| *plsY-*R | ACACGAAAGGTGTTGGTGGT | *W. cibaria* |
| *plsC-*F | GTCCTCACCGTACTTGGTGG | *W. cibaria* |
| *plsC-*R | CGGAAAAAGCATTGGCGTGA | *W. cibaria* |
| *cdsA-*F | TTGCAGCAGCCGTTTACCTA | *W. cibaria* |
| *cdsA-*R | ACGCTTCAAGCCAGACTCAA | *W. cibaria* |
| *gpsA-*F | AAGGTATTGGCTGCTGCGAT | *W. cibaria* |
| *gpsA-*R | CCGAGAAAACATCGCGGTTC | *W. cibaria* |
| *clsA_B-*F | TGGCCGGCATAACAAGATGT | *W. cibaria* |
| *clsA_B-*R | TGGCTTGCGTCCCAAGTATT | *W. cibaria* |
| *glk-*F | TTTCCGTGCCGCATTTGAAG | *E. coli* PR |
| *glk-*R | TAAATGTGCACCGGAACCGA | *E. coli* PR |
| *pgi-*F | AGCTCTGCGTCCGTACAAAA | *E. coli* PR |
| *pgi-*R | GTGATAGCCGGAGCGATGAA | *E. coli* PR |
| *glmS-*F | GTTCACCTGCAGCGTAGGCT | *E. coli* PR |
| *glmS-*R | GACTCTGCGTGAGGCCGTTC | *E. coli* PR |
| *glmM-*F | GCAACATGGGGCTTGAACTG | *E. coli* PR |
| *glmM-*R | TGACCGGAATTCTCTGCACC | *E. coli* PR |
| *glmU-*F | GCTGTTAGCAGGCGTTATGC | *E. coli* PR |
| *glmU-*R | CCGATTTTCACGCGATGACC | *E. coli* PR |
| *murA-*F | AGTCACAATTTCCGGCGCTA | *E. coli* PR |
| *murA-*R | TCGGGACGTTCTGGATCTCT | *E. coli* PR |
| *murB-*F | TGGCAATGCCGGTAGTTTCT | *E. coli* PR |
| *murB-*R | CCTGGGGGTAATTTGGTGCT | *E. coli* PR |
| *murC-*F | GTCGTCATAACGCGCTGAAC | *E. coli* PR |
| *murC-*R | CCAGCGCACGTAAAATAGCC | *E. coli* PR |
| *murD-*F | GCACCGATTGTGGCGATTAC | *E. coli* PR |
| *murD-*R | TCCAGTAGCATCAACGCAGG | *E. coli* PR |
| *murE-*F | TGTGGTGTGTCTTTGGCTGT | *E. coli* PR |
| *murE-*R | GTTCTTCGGTACGCGGGTTA | *E. coli* PR |
| *murF-*F | GACGACTCCTACAACGCCAA | *E. coli* PR |
| *murF-*R | ACCTGTACATGGCAGGCTTC | *E. coli* PR |
| *mraY-*F | CGACGGTCCTGAATCACACT | *E. coli* PR |
| *mraY-*R | CGTACCTTCAGCGTTGCCAG | *E. coli* PR |
| *mrcA-*F | GGACCCGTGGTTTATCAGCA | *E. coli* PR |
| *mrcA-*R | ATTCCGGGCAGGCTACTTTC | *E. coli* PR |
| *mrcB-*F | GACGGCATCAGCCTCTACTC | *E. coli* PR |
| *mrcB-*R | CGCTGGAGAGGAACAGGTTT | *E. coli* PR |
| *mtgA-*F | AACGCTGGGGATAGAAACGG | *E. coli* PR |
| *mtgA-*R | CAAACACACCGTCGCCAAAT | *E. coli* PR |
| *dacC-*F | AAAACTGGGGCTGACCAACA | *E. coli* PR |
| *dacC -*R | GGCACATCGTGGATCAATGC | *E. coli* PR |
| *pfkA-*F | GTGGAAGTGATGGGCCGTTA | *E. coli* PR |
| *pfkA-*R | CCAGGTCTTCACGGCTGAAT | *E. coli* PR |
| *fbaB-*F | TCTTACTGGTGGATCGCGTG | *E. coli* PR |
| *fbaB-*R | ATCAGCACACCCGGGAAAAT | *E. coli* PR |
| *gapA-*F | GACACTCTGGATGGCAACGA | *E. coli* PR |
| *gapA-*R | CGTCATCGTGCCGACTTCTA | *E. coli* PR |
| *pgk-*F | TCGCTGACCAGCTGATTGTT | *E. coli* PR |
| *pgk-*R | TCGTCAACCAGGTCAGCTTC | *E. coli* PR |
| *eno-*F | TGGCGCGAAAACTGTGAAAG | *E. coli* PR |
| *eno-*R | TAGCCACCTTCGTCACCAAC | *E. coli* PR |
| *pyk-*F | CAAAACTGGGGCGTCATGTG | *E. coli* PR |
| *pyk-*R | GTCGCCTTCACCTTTACCCA | *E. coli* PR |
| *aceE-*F | GAGCCAGCGACTGAAACTCT | *E. coli* PR |
| *aceE-*R | GTGATGCCGTAGGTTTTCGC | *E. coli* PR |
| *coaA-*F | CTATTAAGCCGTTGGCCGGA | *E. coli* PR |
| *coaA-*R | CAAACTTCACCAGGCGATGC | *E. coli* PR |
| *coaE-*F | AGACGCAACACCAGATCCAG | *E. coli* PR |
| *coaE-*R | TTTCTGGGCTGACATCCACC | *E. coli* PR |
| *accA-*F | CGTTATCTCGCCGGAAGGTT | *E. coli* PR |
| *accA-*R | TGATACCCATCGCTTCAGCC | *E. coli* PR |
| *accB-*F | ACATCGTACGTTCCCCGATG | *E. coli* PR |
| *accB-*R | ATCGCCCACGTTGACTTTCT | *E. coli* PR |
| *accC-*F | AACGGTCTGTATTGGCCCTG | *E. coli* PR |
| *accC-*R | TCTCGGAGAGGAAGCCGTAA | *E. coli* PR |
| *accD-*F | GCACTGGCAAAAATGCAGGA | *E. coli* PR |
| *accD-*R | GCATGGCGAAACTTGCAGAA | *E. coli* PR |
| *fabD-*F | GTCACAGCCTGGGGGAATAC | *E. coli* PR |
| *fabD-*R | TTCTTGCATGAACTTGCCGC | *E. coli* PR |
| *fabF-*F | TTCTCCGGGTAAAACCTGGC | *E. coli* PR |
| *fabF-*R | CCCACATGCCATAACCCCAT | *E. coli* PR |
| *fabH-*F | AGTTGCTGACGCTGCCTAAT | *E. coli* PR |
| *fabH-*R | GCGTCTCATCAACGATGTGC | *E. coli* PR |
| *fabG-*F | TAACGATCTTCCCGATGCCG | *E. coli* PR |
| *fabG-*R | ATCGGCAGGGAAGCAAATCA | *E. coli* PR |
| *fabZ-*F | TCTTACTGGTGGATCGCGTG | *E. coli* PR |
| *fabZ-*R | ATCAGCACACCCGGGAAAAT | *E. coli* PR |
| *fabI-*F | AAAGCGTCTCTGGAAGCGAA | *E. coli* PR |
| *fabI-*R | GATCGGACCAGCAGAGATGG | *E. coli* PR |
| *plgK-*F | TAAGCTGTTTATGGCGGGCA | *E. coli* PR |
| *plgK-*R | TGCCAGATTTCCTGCATCGT | *E. coli* PR |
| *plsX-*F | TCATGGGAGGGGATTTTGGC | *E. coli* PR |
| *plsX-*R | CAAGTAATGGCGTGATGGCG | *E. coli* PR |
| *plsY-*F | GGGAACCTGGTTACTGACCG | *E. coli* PR |
| *plsY-*R | CATCGAAACCGGGAAGGTGA | *E. coli* PR |
| *plsC-*F | GCCTGCTACCGTTCAAGACT | *E. coli* PR |
| *plsC-*R | AGTTGTAGAGACGCACACGG | *E. coli* PR |
| *ynbB-*F | ACCACAAATATCCAGGGCGG | *E. coli* PR |
| *ynbB-*R | GGGCTGACTTTAGGGACCAC | *E. coli* PR |
| *cdsA-*F | TTCTCCGGGTAAAACCTGGC | *E. coli* PR |
| *cdsA-*R | CCCACATGCCATAACCCCAT | *E. coli* PR |
| *pgsA-*F | CAGCTGGTGGGTGACCTTAC | *E. coli* PR |
| *pgsA-*R | ACGCTACTGCGTTTACCCAA | *E. coli* PR |
| *cls-*F | CAGTCGCGCATTCTTTACGG | *E. coli* PR |
| *cls-*R | ACGGTGCCAACCAGACTTAG | *E. coli* PR |
| *clsB-*F | GGAAGTCTTGCTGGATGGCT | *E. coli* PR |
| *clsB-*R | CGCATACCAAAAAGGCGAGG | *E. coli* PR |

**TABLE S3** Primers of genes related to biofilm formation and cell division

| Pimer | Sequence（5’ - 3’） | Bacterial strain |
| --- | --- | --- |
| *glgA-*F | GTGCTTATGCAGCAGACACG | *W. cibaria* |
| *glgA-*R | CCATCCCCGTCTTCAACCAA | *W. cibaria* |
| *glgC-*F | CGAGTCACCGTGAACAGGAA | *W. cibaria* |
| *glgC-*R | CCAGCATTCCTGGCAACAAC | *W. cibaria* |
| *glgP-*F | TCCAACGGTGGTCATTCCAG | *W. cibaria* |
| *glgP-*R | TCCAAAGCTTCCGCTAGCAA | *W. cibaria* |
| *pgaC-*F | TTTGCAGGGGATGTAGACCG | *W. cibaria* |
| *pgaC-*R | TCGGCCGAAACCATTACCAA | *W. cibaria* |
| *wecB-*F | TCCAGTTGGTCACGTTGAGG | *W. cibaria* |
| *wecB-*R | GCCAAGTTATTCGCGCTCAG | *W. cibaria* |
| *dinJ-*F | CGTAATGCAGCCGATGAGGT | *W. cibaria* |
| *dinJ-*R | TCCGTTATTAGCCACCTGTGT | *W. cibaria* |
| *secA-*F | AACCGACCTGTGCAACGTAT | *W. cibaria* |
| *secA-*R | TGACCCTTCTCGTGCAGTTC | *W. cibaria* |
| *secE-*F | TTTAGCAAGCGTTGTCGCTG | *W. cibaria* |
| *secE-*R | GCCACCAAACAAAATGGCGA | *W. cibaria* |
| *secG-*F | CGATGATTGTCGTTGGCGTG | *W. cibaria* |
| *secG-*R | GTTGCATCACTGCCTCGAAC | *W. cibaria* |
| *secY-*F | CGGGAACAATGTTTGCCGTT | *W. cibaria* |
| *secY-*R | CACACGCGCAATGATACCAG | *W. cibaria* |
| *yajC-*F | TGAAGCACCCAAGGCAGAAA | *W. cibaria* |
| *yajC-*R | AGCGTCGTCTGACTTTTCGT | *W. cibaria* |
| *yidC-*F | GGTGGTTTCAAACGCCTTCC | *W. cibaria* |
| *yidC-*R | ATCACGCTCTGCTTGCTTCT | *W. cibaria* |
| *dnaA-*F | ACGACGTCCAATTCTTGGCT | *W. cibaria* |
| *dnaA-*R | GCGTGACCAAACGCATTTCT | *W. cibaria* |
| *dnaB-*F | CGACAGAAACTGTGACGGGA | *W. cibaria* |
| *dnaB-*R | CAGCCACCTTCTGGGCAATA | *W. cibaria* |
| *ftsA-*F | TGAAGGGGGTCGCCTATACA | *W. cibaria* |
| *ftsA-*R | GCAAGTGGCGCTAACACAAA | *W. cibaria* |
| *ftsQ-*F | GGCGGCATTCTTAGGCTACT | *W. cibaria* |
| *ftsQ-*R | ATCCAGCCGCCTTTAAGACC | *W. cibaria* |
| *ftsW-*F | GCATGCAGTACCGCTTGATG | *W. cibaria* |
| *ftsW-*R | GCAGTAATCCGACAACCCCA | *W. cibaria* |
| *ftsZ-*F | ACCAAATGGTGAGCGATGGT | *W. cibaria* |
| *ftsZ-*R | CACGAGTTGCCTTTGTTCCG | *W. cibaria* |
| *murG-*F | GTGCGCTGAAGCTGAACAAA | *W. cibaria* |
| *murG-*R | TTCATCAAAGCGCTTGCCAC | *W. cibaria* |
| *rseP-*F | CGTGCTGAGGCTGAAGAAGA | *W. cibaria* |
| *rseP-*R | TAACGGTTAACGCACGACCA | *W. cibaria* |
| *glgA-*F | CGCCAGTGGGTTTGTCTTTG | *E. coli* PR |
| *glgA-*R | CACAAACCGCCACAGTGAAG | *E. coli* PR |
| *glgC-*F | GCGGGCGACCATATCTACAA | *E. coli* PR |
| *glgC-*R | CATAACGCCAAATGCGGAGG | *E. coli* PR |
| *pgaC-*F | CATACACGCCGCTTTAGCAC | *E. coli* PR |
| *pgaC-*R | ATGACCCGCAAATGGGGAAT | *E. coli* PR |
| *wecB-*F | AGCAGCGAATTGTCGAGGAA | *E. coli* PR |
| *wecB-*R | AACGCTTCCAGAATGCGAGA | *E. coli* PR |
| *wecC-*F | GACGGCGGAAATGTGTAAGC | *E. coli* PR |
| *wecC-*R | GATCGGCACAAATCAGCGAC | *E. coli* PR |
| *bcsA-*F | CAGGGAAGAGTTTCGCCAGT | *E. coli* PR |
| *bcsA-*R | TACGTGGTCGCAGTCGAAAA | *E. coli* PR |
| *csgA-*F | TCTGGCAGGTGTTGTTCCTC | *E. coli* PR |
| *csgA-*R | CCGCCACCGTACTGGTAAAT | *E. coli* PR |
| *csgB-*F | GCCAGTATTTCGCAAGGTGC | *E. coli* PR |
| *csgB-*R | GTTGTGTCACGCGAATAGCC | *E. coli* PR |
| *csgC-*F | TACTCCTTGCGGCACTTTCC | *E. coli* PR |
| *csgC-*R | ACTTTGCCCTGAACTGCCTT | *E. coli* PR |
| *csgD-*F | CGCGGCGAATGCTACTTTAC | *E. coli* PR |
| *csgD-*R | TCGTTATTAGACGCGCCGAT | *E. coli* PR |
| *secA-*F | GGAAAAACCCTGACCGCAAC | *E. coli* PR |
| *secA-*R | CGACAGTCAGGCCAAGGAAT | *E. coli* PR |
| *secB-*F | TTCCCGTATGCTCGTGAGTG | *E. coli* PR |
| *secB-*R | TTCCCGTATGCTCGTGAGTG | *E. coli* PR |
| *secD-*F | CTCGTGATGAAGCCATTGCG | *E. coli* PR |
| *secD-*R | CTGCTGCACCGCATATTCAC | *E. coli* PR |
| *secE-*F | CTCGCCAGGAAACATTGCAC | *E. coli* PR |
| *secE-*R | ATCCAGTCCCCACAGGATCA | *E. coli* PR |
| *secF-*F | GGCATCTCTGGTCTGCTGTT | *E. coli* PR |
| *secF-*R | TACCACCGGTGAAATCCAGC | *E. coli* PR |
| *secG-*F | TGTGGCAATTGGCCTTGTTG | *E. coli* PR |
| *secG-*R | AACCAAACAGCGTAGCGGAA | *E. coli* PR |
| *secM-*F | CTTGCTGGAAGCGAACACAC | *E. coli* PR |
| *secM-*R | AGATTCTTCAGCAACGGGCA | *E. coli* PR |
| *secY-*F | CCTCGTGTTGCTGTTGGTTG | *E. coli* PR |
| *secY-*R | CTTGCTGACGTTTCGCGTAG | *E. coli* PR |
| *yajC-*F | GAAGCGCACCAAAGAACACA | *E. coli* PR |
| *yajC-*R | CAGCGCGATAGCAATGTAGC | *E. coli* PR |
| *yidC-*F | GAGCGTCTGGGTGATGACAA | *E. coli* PR |
| *yidC-*R | AACGGAACCCATCAGCATGT | *E. coli* PR |
| *dnaA-*F | GACTGACTGTGGCGATCGAA | *E. coli* PR |
| *dnaA-*R | TGGCGATAAAGAACGCCACT | *E. coli* PR |
| *dnaB-*F | CAAACAAAGACGAAGGGCCG | *E. coli* PR |
| *dnaB-*R | GGCTGCTGAAACAACTGCTC | *E. coli* PR |
| *ftsA-*F | CCGTCGCGTGGTATGGATA | *E. coli* PR |
| *ftsA-*R | TGCCATCAATTCTGCCTGGT | *E. coli* PR |
| *ftsQ-*F | CTCGCCGCAATAATGGAACG | *E. coli* PR |
| *ftsQ-*R | TCTTCCATCCAGCCCAACAC | *E. coli* PR |
| *ftsW-*F | GGCATTTCAGCGGTTGTGTT | *E. coli* PR |
| *ftsW-*R | TTCCCACGGGTTCCAGAATG | *E. coli* PR |
| *murG-*F | GACTACCGGCGTTGTTTGTG | *E. coli* PR |
| *murG-*R | CTAAGCTGTGGCTGCTCGAT | *E. coli* PR |
| *rseP-*F | TTACGTCACTGGGCGTTTGA | *E. coli* PR |
| *rseP-*R | CTTGCAAACCTGCCTTGCTT | *E. coli* PR |
